# Identification of a small molecule that primes the type I interferon response to cytosolic DNA

**DOI:** 10.1038/s41598-017-02776-z

**Published:** 2017-05-31

**Authors:** Samira Khiar, Marianne Lucas-Hourani, Sébastien Nisole, Nikaïa Smith, Olivier Helynck, Maryline Bourgine, Claude Ruffié, Jean-Philippe Herbeuval, Hélène Munier-Lehmann, Frédéric Tangy, Pierre-Olivier Vidalain

**Affiliations:** 1 Unité de Génomique Virale et Vaccination, Institut Pasteur, CNRS UMR3569, Paris, France; 2 Mécanismes d’action des interférons et voies bio-thérapeutiques, Université Paris Descartes, INSERM UMR-S1124, Paris, France; 3 Chimie & Biologie, Modélisation et Immunologie pour la Thérapie (CBMIT), Université Paris Descartes, CNRS UMR8601, Paris, France; 4 Unité de Chimie et Biocatalyse, Institut Pasteur, CNRS UMR3523, Paris, France; 5 Unité de Virologie Moléculaire et Vaccinologie, Institut Pasteur, Paris, France

## Abstract

The type I interferon response plays a pivotal role in host defense against infectious agents and tumors, and promising therapeutic approaches rely on small molecules designed to boost this system. To identify such compounds, we developed a high-throughput screening assay based on HEK-293 cells expressing luciferase under the control of Interferon-Stimulated Response Elements (ISRE). An original library of 10,000 synthetic compounds was screened, and we identified a series of 1H-benzimidazole-4-carboxamide compounds inducing the ISRE promoter sequence, specific cellular Interferon-Stimulated Genes (ISGs), and the phosphorylation of Interferon Regulatory Factor (IRF) 3. ISRE induction by ChX710, a prototypical member of this chemical series, was dependent on the adaptor MAVS and IRF1, but was IRF3 independent. Although it was unable to trigger type I IFN secretion *per se*, ChX710 efficiently primed cellular response to transfected plasmid DNA as assessed by potent synergistic effects on IFN-β secretion and ISG expression levels. This cellular response was dependent on STING, a key adaptor involved in the sensing of cytosolic DNA and immune activation by various pathogens, stress signals and tumorigenesis. Our results demonstrate that cellular response to cytosolic DNA can be boosted with a small molecule, and potential applications in antimicrobial and cancer therapies are discussed.

## Introduction

Defense mechanisms against infectious agents and tumors critically rely on type I interferon (IFN) response^1^. This system is based on the induction of both IFN-α/β cytokines and Interferon-Stimulated Genes (ISGs), which both contribute to the activation of innate and adaptive immunity^2^. Type I IFN response is triggered by the recognition of specific Pathogen-Associated Molecular Patterns (PAMPs) and Damage-Associated Molecular Patterns (DAMPs). PAMPs correspond to a limited set of molecular structures, including proteins, sugars, lipids or nucleic acids, which are specifically associated to infectious agents, whereas DAMPs originate from injured or dead cells^3^. Different classes of cellular receptors, known as PRR for “Pattern Recognition Receptors”, are involved in the recognition of PAMPs and DAMPs. Key PRRs involved in the induction of type I IFN response are toll-like receptors 3, 7, 8 or 9 (TLR3/7/8/9), RIG-I like receptors (RLRs) that signal through the adaptor MAVS, and cytosolic DNA sensors such as cGAS or IFI16 that signal by using STING as a platform^4, 5^. These different receptors essentially recognize nucleic acids with unusual features or localization patterns such as 5′-triphosphate RNA molecules for RIG-I or cytosolic DNA for the cGAS/STING signaling pathway. Once engaged by their ligands, they activate Interferon Regulatory Factors (IRF3, IRF7 or IRF1) to induce the expression of type I IFN cytokines (IFN-α and β) together with a first set of genes usually referred as early ISGs^1^. Later on, secreted IFN-α/β bind to their membrane receptor at the surface of both IFN-producing and neighboring cells to amplify the immune response. IFN-α/β binding to their receptor activates STAT1 and STAT2 transcription factors and a second wave of ISGs to further control the infection or eliminate tumor cells^6^. It is estimated that human genome contains hundreds of ISGs that, for example, interfere with virus replication, sensitize tumor cells to apoptosis, or stimulate the adaptive immune response^1, 2, 7^. Deciphering the complex mechanisms regulating the type I IFN response is essential to the development of innovative therapies that stimulate the immune system against infections or tumors, without leading to overactivation of this system which can be deleterious for the patient^8^.

The quest for small compounds activating the type I interferon response is a field of intense researches in academic laboratories and pharmaceutical companies^9–18^, and some molecules are already marketed or in advanced clinical trials^19, 20^. Compounds from imidazoquinoline family, such as resiquimod (R848) and imiquimod (R837), are well-known inducers of the IFN response that bind TLR7, TLR8 or both^21^. Pyrimidine and purine derivatives have been also characterized as TLR7/8 ligands, and are currently in development. More recently, it has been shown that 5,6-di-methylxanthenone-4-acetic acid (DMXAA), 10-carboxymethyl-9-acridanone (CMA), and flavone acetic acid (FAA), which have all been known for many years to induce the type I IFN response in mice but not human, are specific ligands of mouse STING^22–25^. STING is a signaling protein that is activated by multiple cytoplasmic proteins involved in DNA sensing, and was even proposed to bind DNA directly, but is also a receptor for 2′,3′-cGAMP, an endogenous cyclic dinucleotide produced by the cellular enzyme cGAS in the presence of cytosolic DNA^4^. DMXAA, CMA and FAA bind specifically mouse STING in the 2′,3′-cGAMP binding pocket, thus triggering IRF3 activation and the induction of ISGs. Based on these results, several research groups are looking for small compounds capable of binding human STING^26, 27^. Finally, compounds directly binding the type I IFN receptor to induce ISGs were also reported^28^.

We previously described a high-throughput cellular assay that can be used to screen chemical libraries, and identify molecules inducing ISGs^29^. This screening system is based on a HEK-293 cell line expressing luciferase under control of five Interferon-Stimulated Response Elements (ISRE). These regulatory elements are present in the promoters of all ISGs to ensure gene activation upon STAT1/2 activation, but also contain binding sites for IRF1/3/7, which are directly activated by PRRs. Luciferase activity is used as read-out to select molecules that induce ISGs. Since HEK-293 cells do not express significant levels of TLR3, 7, 8 or 9^30^, this assay essentially selects for ligands of type I IFN receptors, activators of STING or RLR pathways, or yet unidentified pathways. For example, we previously identified with this assay different compound families targeting *de novo* pyrimidine biosynthesis through inhibition of dihydroorotate dehydrogenase (DHODH), the fourth enzyme of this metabolic pathway^29^. This led us to establish for the first time a functional link between the interferon response and the cellular stress induced by depleting pools of pyrimidines.

To capture new immunostimulatory molecules with a different mode of action, we screened an original chemical library of 10,000 compounds using the ISRE-luciferase reporter system described above. This allowed the identification of an original molecule amplifying the type I IFN response to transfected DNA along a STING-dependent pathway. Altogether, these results demonstrate that type I IFN response to exogenous DNA can be boosted with a small molecule, which should help the development of novel treatments based on manipulations of the immune response against infections and tumors.

## Results

### ChX67779 induces the ISRE-luciferase reporter gene

To identify compounds that stimulate the expression of ISGs, a library of 10,000 chemically diverse molecules was screened with a high-throughput cellular assay based on HEK-293 cells expressing luciferase under control of five ISRE copies (STING-37 reporter cell line; see Materials and Methods for details). Cells were cultured in the presence of tested compounds at a final concentration of 20 μM, and luciferase activity was revealed by addition of a commercial luciferin-based reagent after 24 hours of stimulation. In positive control wells of each screening plate, an average 77-fold induction of the luciferase signal was observed in the presence of recombinant IFN-β. Of the 10,000 molecules tested, only two related 1H-benzimidazole-4-carboxamide derivatives scored positive with >3-fold inductions of the ISRE-luciferase reporter gene: ChX0275199 and ChX67779, which is more active and was selected for further characterizations (Supp. Table 1; top rows). We first determined if this activity was linked to the inhibition of DHODH, since others and we previously reported that compounds targeting this enzyme activate the ISRE promoter sequences^29, 31–33^. When culture medium was supplemented with uridine to reverse-complement for the inhibition of DHODH, ISRE-luciferase induction by ChX67779 was maintained (data not shown), thus excluding that ChX67779 targets this enzyme. We then decided to further characterize biological properties of ChX67779 as an original activator of ISRE sequences.

**Figure 1:**
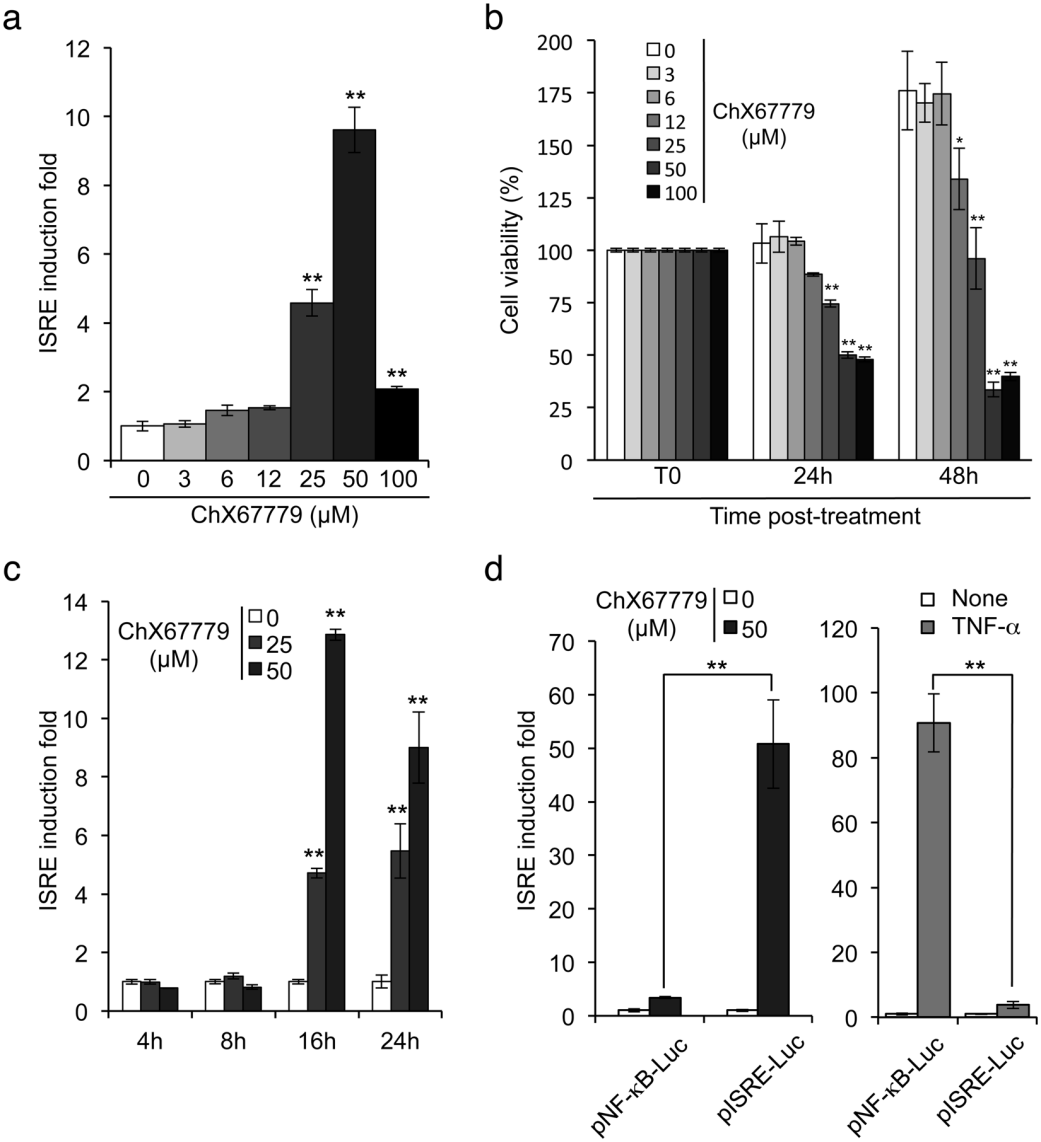
ChX67779 activates the ISRE-luciferase reporter gene. (**a**) HEK-293 cells expressing luciferase under control of five interferon-stimulated response elements (ISRE) were incubated, at 4 × 10^4^ cells/well in a 96-well plate, with DMSO alone or increasing concentrations of ChX67779 ranging from 3 to 100 μM. After 24 hours of incubation, luciferase induction was determined. (**b**) HEK-293 cells were incubated with increasing concentrations of ChX67779 as described above. After 0, 24 and 48 hours of culture, the number of metabolically active cells was determined by ATP quantification using the CellTiter-GLO reagent. Results are expressed as a percentage relative to the initial number of living cells at t = 0 hour. (**c**) ISRE-luciferase reporter cells were left untreated (DMSO alone) or incubated with ChX67779 at 25 or 50 μM. Luciferase induction was determined after 4, 8, 16 and 24 hours of culture. (**d**) HEK-293T cells were transfected with pNF-κB-Luc or pISRE-Luc using 50 ng plasmid for 4 × 10^4^ cells/well in a 96-well plate. Cells were treated with ChX67779 at 50 μM or TNF-α at 10 ng/ml. After 24 hours of incubation, luciferase induction was determined. Experiments were performed in triplicate, and data represent means ± SD. *P < 0.05 and **P < 0.01 as calculated by one-way ANOVA with Tukey’s post hoc test (**a** and **b**), two-way ANOVA with Bonferroni’s post hoc test (**c**), or Student’s t test (**d**).

First, ChX67779 was retested for the induction of the ISRE-luciferase reporter gene in a dose-response experiment. As shown in Fig. 1a, a 10-fold induction peak was observed at 50 μM, but luciferase activity declined at 100 μM, suggesting some toxicity of the molecule and the collapse of cellular functions at highest concentrations. Viability of ChX67779-treated cells was determined after 24 or 48 hours of treatment by quantifying ATP in culture wells, which reflects the metabolic activity of the cells (Fig. 1b). ChX67779 treatment decreased cellular ATP levels in culture wells in dose-dependent manner, thus demonstrating the significant toxicity of this molecule. Alteration of cellular viability in cultures was also confirmed by bright-field microscopy (data not shown). In a kinetic experiment, we found that ISRE-luciferase induction by ChX67779 occurred at relatively late time-points, between 8 and 16 hours of treatment (Fig. 1c). Most importantly, we tested if ChX67779 activity was specific of the ISRE promoter sequence by transient transfection of HEK-293T cells with either ISRE-luciferase or NF-κB-luciferase reporter constructs. As shown in Fig. 1d, ChX67779 only induced the ISRE-luciferase reporter gene, but not the NF-κB-luciferase reporter gene, which was specifically activated by recombinant TNF-α. Interestingly, ISRE-luciferase induction by ChX67779 was much more pronounced in transiently transfected cells compared to stable cell line (compare Fig. 1a and d). Altogether, our results demonstrate that ChX67779 specifically stimulates ISRE promoter sequences but also exhibits some significant cellular toxicity.

### Induction of ISGs by ChX67779 in different cell lines

**Figure 2:**
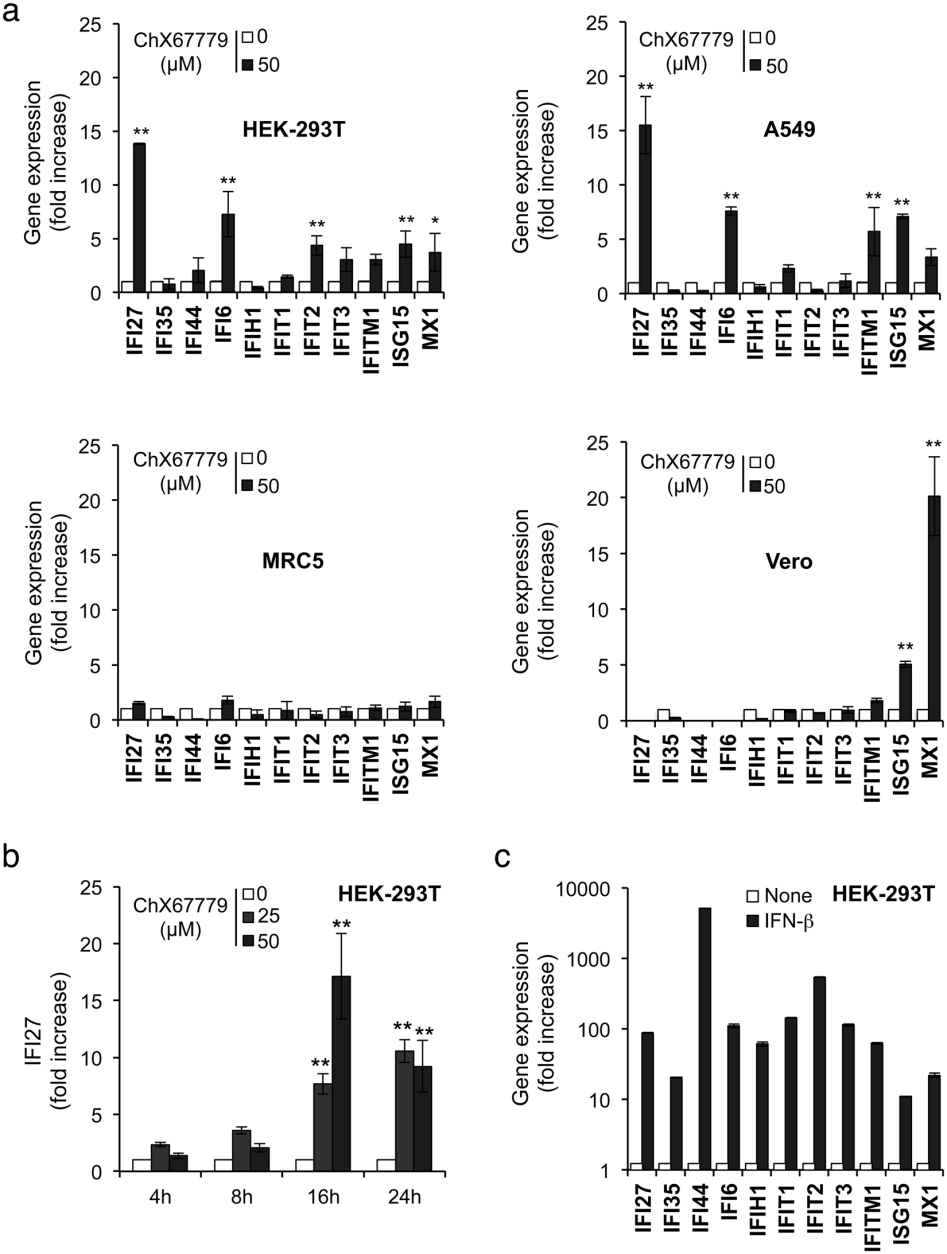
Induction of IFN-inducible genes (ISGs) by ChX67779. (**a**) HEK-293T, A549, MRC5 and Vero cells at 2 × 10^5^ cells/well in 24-well plates were stimulated with DMSO alone or ChX67779 at 50 μM. After 24 hours of culture, total RNAs were extracted, and expression levels of indicated genes were quantified by qRT-PCR. Data were normalized to the average expression of four housekeeping genes (18 S, GUSB, GAPDH, and HPRT1), and expressed as induction folds relative to DMSO-treated cells using the 2^−ΔΔCt^ method. (**b**) HEK-293T cells were left untreated (DMSO alone) or incubated with ChX67779 for 0, 4, 16 or 24 hours. IFI27 induction was determined by RT-qPCR as described above. (**c**) HEK-293T cells were left untreated or stimulated with recombinant IFN-β (500 IU/ml) for 24 hours. ISG expression levels were determined by RT-qPCR as described above. Experiments were performed in duplicate, and data represent means ± SD. *P < 0.05 and **P < 0.01 as calculated by two-way ANOVA with Bonferroni’s post hoc test.

We then determined if ChX67779 could not only induce the ISRE-luciferase reporter gene but also cellular ISGs. Different cell types, including HEK-293T (embryonic kidney epithelial cells; human), A549 (lung epithelial adenocarcinoma; human), MRC5 (lung fibroblasts; human) and Vero cells (kidney epithelial cells; African green monkey) were treated with ChX67779 at 50 μM for 24 hours, and expression levels of a panel of 11 well-characterized ISGs were determined by RT-qPCR. As shown in Fig. 2a, ChX67779 induced some ISGs in HEK-293T cells, in particular IFI27 and IFI6, while others were not activated. Similar results were obtained in A549 cells. In contrast, the induction profile was very different in Vero cells where ISG15 and Mx1 were essentially induced, whereas ChX67779 treatment had no effect on tested ISGs in MRC5 cells. These results confirmed ChX67779 capacity to specifically induce some ISGs with a profile that clearly depends on the cell type considered. Interestingly, ChX67779 was relatively less toxic to MRC5 compared to other cell lines at 50 μM (Supp. Fig. 1), suggesting that cytoxicity could be linked to the induction of ISGs. A kinetic analysis of IFI27 expression levels confirmed that ChX67779 induced ISGs between 8 and 16 hours post-treatment (Fig. 2b), in agreement with results obtained with the ISRE-luciferase reporter gene (Fig. 1c). We also compared ChX67779 to recombinant IFN-β with respect to its capacity to stimulate ISGs. As shown in Fig. 2c, IFN-β induced the expression of all tested ISGs much more efficiently than ChX67779, and without any detectable cytotoxicity. Altogether, these results confirm ChX67779′s capacity to induce some ISGs but by comparison with IFN-β, suggest that a signaling pathway different from the canonical JAK/STAT cascade is engaged.

### Establishing structure/activity relationships with chemical analogs of ChX67779

**Figure 3:**
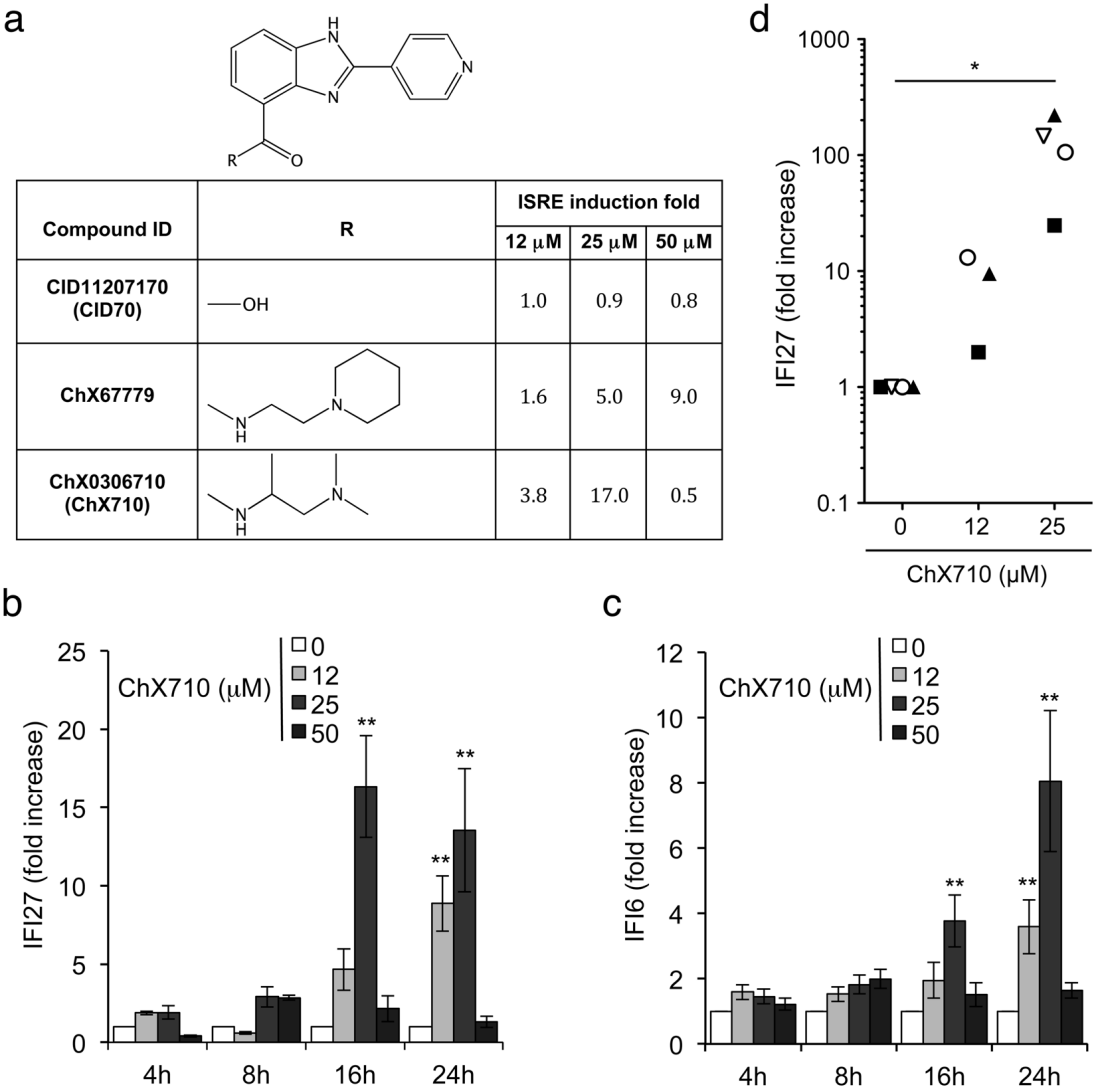
Structure activity relationships and selection of ChX710. (**a**) Chemical structures of ChX67779 and two analogs called CID11207170 and ChX0306710. Compounds were tested for their capacity to induce the ISRE-luciferase reporter gene at three different concentrations. (**b** and **c**) HEK-293T cells were left untreated (DMSO alone) or incubated with ChX710 at indicated concentrations for 0, 4, 16 or 24 hours. IFI27 (**b**) and IFI6 (**c**) induction levels were determined by RT-qPCR as described above. Experiments were performed in duplicate, and data represent means ± SD. *P < 0.05 and **P < 0.01 as calculated by two-way ANOVA with Bonferroni’s post hoc test. (**d**) Human PBMCs were treated for 24 hours with DMSO alone or ChX710 at 12.5 or 25 μM, and IFI27 inductions was determined by RT-qPCR as described above. Data correspond to four healthy donors depicted by different symbols. *P < 0.05 as calculated by one-way ANOVA with Bonferroni’s post hoc test.

To demonstrate that ChX67779 biological activity is linked to its chemical structure (Fig. 3a), we analyzed a set of 50 analogs that were commercially available, and determined structure/activity relationships. We first tested numerous substitutions on the carboxamide group. A compound in which the carboxamide was replaced by a carboxylic acid was totally inactive (CID11207170, referred as CID70), supporting the key role played by this part of the molecule (Fig. 3a). Interestingly, this inactive compound showed a very limited impact on cellular viability (Supp. Fig. 2a), further supporting a link between the induction of ISGs by ChX67779 and its cellular toxicity. Then, we tested substitutions of the aminoethylpiperidine group of ChX67779 by various amines (Supp. Table 1). Most of them were totally inactive, except few analogs including ChX0275199 that was a hit in the initial screen. Interestingly, ChX0306710 (referred as ChX710) was significantly more active than ChX67779 with a peak of induction at 25 μM (Fig. 3a). In contrast, the activity was lost when replacing the pyridine moiety by phenol or dimethylaniline moieties (Supp. Table 2), thus demonstrating the importance of this heterocycle. Finally, we confirmed that the most active analog identified (ChX710) also induced some ISGs as assessed by measuring the induction of IFI27 and IFI6 in HEK-293T treated with this molecule (Fig. 3b and c). IFI27 induction was also established in peripheral blood mononuclear cells (PBMC) from healthy donors when treated with 12 or 25 μM of ChX710, thus supporting its activity in primary human cells (Fig. 3d). However, the viability of PBMCs was deeply altered when ChX710 was applied at a higher concentration (50 μM; Supp. Fig. 2c). We thus decided to further investigate the signaling pathways involved using ChX710 as a prototype of this chemical series.

### ISRE induction by ChX710 depends on MAVS and IRF1

**Figure 4:**
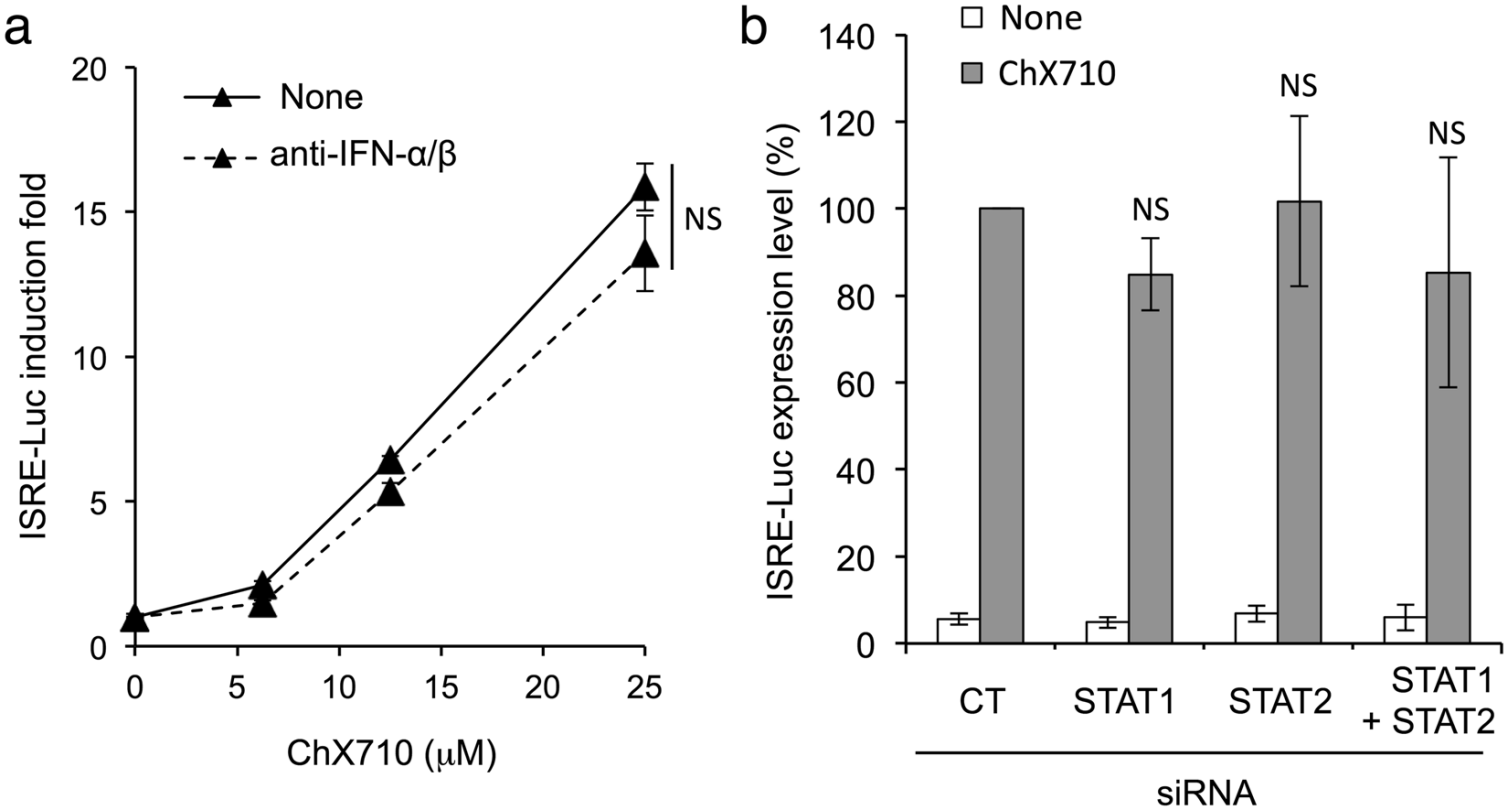
ISRE induction by ChX710 is independent of IFN-α/β, STAT1, and STAT2. (**a**) ISRE-luciferase reporter cells were incubated, at 4x10^4^ cells/well in a 96-well plate, with DMSO alone or increasing concentrations of ChX710 in the presence of both blocking antibodies against IFN-α/β. After 24 hours of incubation, luciferase induction was determined. Experiment was performed in duplicate, and data represent means ± SD. NS indicates non-significant differences as calculated by two-way ANOVA. (**b**) STAT1, STAT2 or STAT1 and STAT2 were silenced by siRNA transfection in ISRE-luciferase reporter cells. After 48 hours of cultures, cells were stimulated with ChX710. After 24 hours of incubation, luciferase induction was determined. Data represent means ± SD of three independent experiments. NS indicates non-significant differences relative to control siRNA (CT) as calculated by two-way ANOVA.

First, we determined whether type I interferons (IFN-α/β) are involved in the activation of the ISRE regulatory sequence by ChX710. To address this question, ISRE-luciferase reporter cells were incubated with ChX710 in the presence of antagonist antibodies blocking the interaction of IFN-α/β with their cognate receptor IFNAR1/IFNAR2c. Antibody concentrations we used were sufficient to block up to 500 IU/ml of IFN-α or β. Nevertheless, no impact on ISRE-luciferase induction by ChX710 was detected (Fig. 4a). Furthermore, we showed by RT-qPCR that Chx710-treatment does not significantly induce IFN-α, IFN-β, IFN-γ or IFN-λ mRNA transcripts (see below). Thus, ISRE-luciferase activation by ChX710 is independent of some autocrine/paracrine loop involving IFN-α/β. We also evaluated the role of STAT1 and STAT2, which are activated downstream of IFN-α/β receptor to induce ISGs. First, we suppressed STAT1 or STAT2 expression by siRNA, and stimulated ISRE-luciferase reporter cells with ChX710. As shown in Fig. 4b, ISRE-luciferase induction was unaffected by the silencing of STAT1, STAT2 or STAT1 + STAT2, despite some efficient suppression of STAT1 and STAT2 expression as assessed by western-blot analysis (Supp. Fig. 3a). As a control, we showed that STAT2 silencing efficiently inhibited ISRE-luciferase induction by recombinant IFN-β (Supp. Fig. 3b), whereas STAT1 was clearly facultative, in agreement with previous reports^34^. Altogether, our results demonstrate that ISRE activation by ChX710 is independent of STAT1 and STAT2.

**Figure 5:**
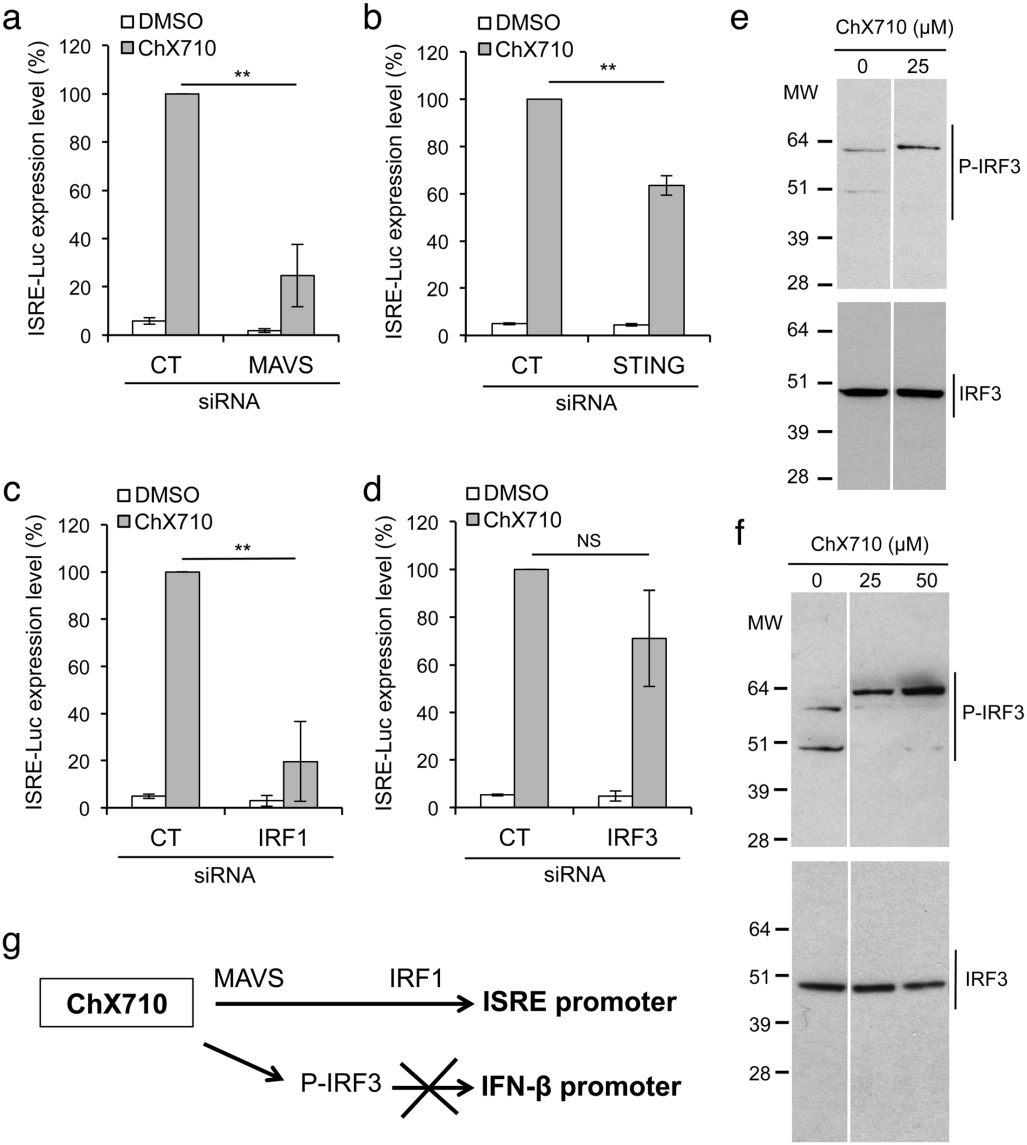
ISRE induction by ChX710 is dependent on MAVS and IRF1. (**a**) MAVS was silenced by siRNA transfection in ISRE-luciferase reporter cells. After 48 hours of cultures, cells were stimulated with ChX710. After 24 hours of incubation, luciferase induction was determined. (**b**) Same experiment as (**a**) using STING-specific siRNA. (**c**) Same experiment as (**a**) using IRF1-specific siRNA. (**d**) Same experiment as (**a**) using IRF3-specific siRNA. (**e,f**) IRF3 phosphorylation was determined by western-blot in HEK-293T (**e**) or A549 cells (**f**) treated for 24 hours with DMSO alone or ChX710 at 25 μM or 50 μM. (**g**) Schematic model of the signaling pathways induced by ChX710. IRF3 phosphorylation is induced, but this is insufficient to activate IFN-β expression. Data represent means ± SD of three independent experiments, except for (**e**) and (**f**) that corresponds to representative results. **P < 0.01 as calculated by Student’s t test.

This suggested some direct activation of the ISRE promoter by intrinsic pathways, possibly involving MAVS or STING signaling pathways together with downstream IRF transcription factors. To test this, we first suppressed MAVS or STING expression by siRNA and stimulated ISRE-luciferase reporter cells with ChX710. As shown in Fig. 5a, ISRE-luciferase induction was suppressed by MAVS silencing, thus involving this adaptor protein in the signaling cascade activated by ChX710. Compared to MAVS, STING silencing only had a modest, although statistically significant, effect on ISRE induction by ChX710 (Fig. 5b). We controlled that MAVS or STING were efficiently silenced, both by western-blot analysis (Supp. Fig. 4a), and showing that ISRE-luciferase inductions by short synthetic 5-triphosphate RNA molecules (ssRNA) or cGAMP were inhibited, respectively (Supp. Fig. 4b and c). We then determined the role of IRF1 and IRF3, two transcription factors involved in the induction of ISGs and interferon genes^1^. We controlled by western-blot analysis that IRF1 or IRF3 were efficiently silenced (Supp. Fig. 4a). In HEK-293 cells, we have previously shown that IRF1 is essential to ISRE-luciferase activation by ssRNA^29^, but that IRF3 is dispensable (Supp. Fig. 4d). In contrast, IRF3 is necessary for the induction of IFN-β promoter by ssRNA (Supp. Fig. 4e), thus defining two different pathways downstream of MAVS that regulate ISGs and interferon genes (Supp. Fig. 4f)^29^. As shown in Fig. 5c, IRF1 silencing impaired ISRE activation by ChX710, whereas IRF3 suppression had no significant effect (Fig. 5d). Nevertheless, ChX710 induced IRF3 phosphorylation in HEK-293T (Fig. 5e) and A549 cells (Fig. 5f), but this was insufficient to trigger IFN-β expression as previously shown. Interestingly, P-IRF3 detected in ChX710-treated cells showed a high molecular weight (about 62 kDa) compared to total IRF3 (about 48 kDa) or basal P-IRF3 detected in untreated cells (two faint bands at about 48 and 60 kDa). This suggests that other post-translational modifications, such as ISGylation for example^35^, affect P-IRF3 in ChX710-treated cells. Altogether, our results demonstrate that a MAVS/IRF1 signaling axis is required for ISRE induction by ChX710, and that IRF3 phosphorylation is induced (Fig. 5g).

### ChX710 primes the interferon response to foreign DNA

**Figure 6:**
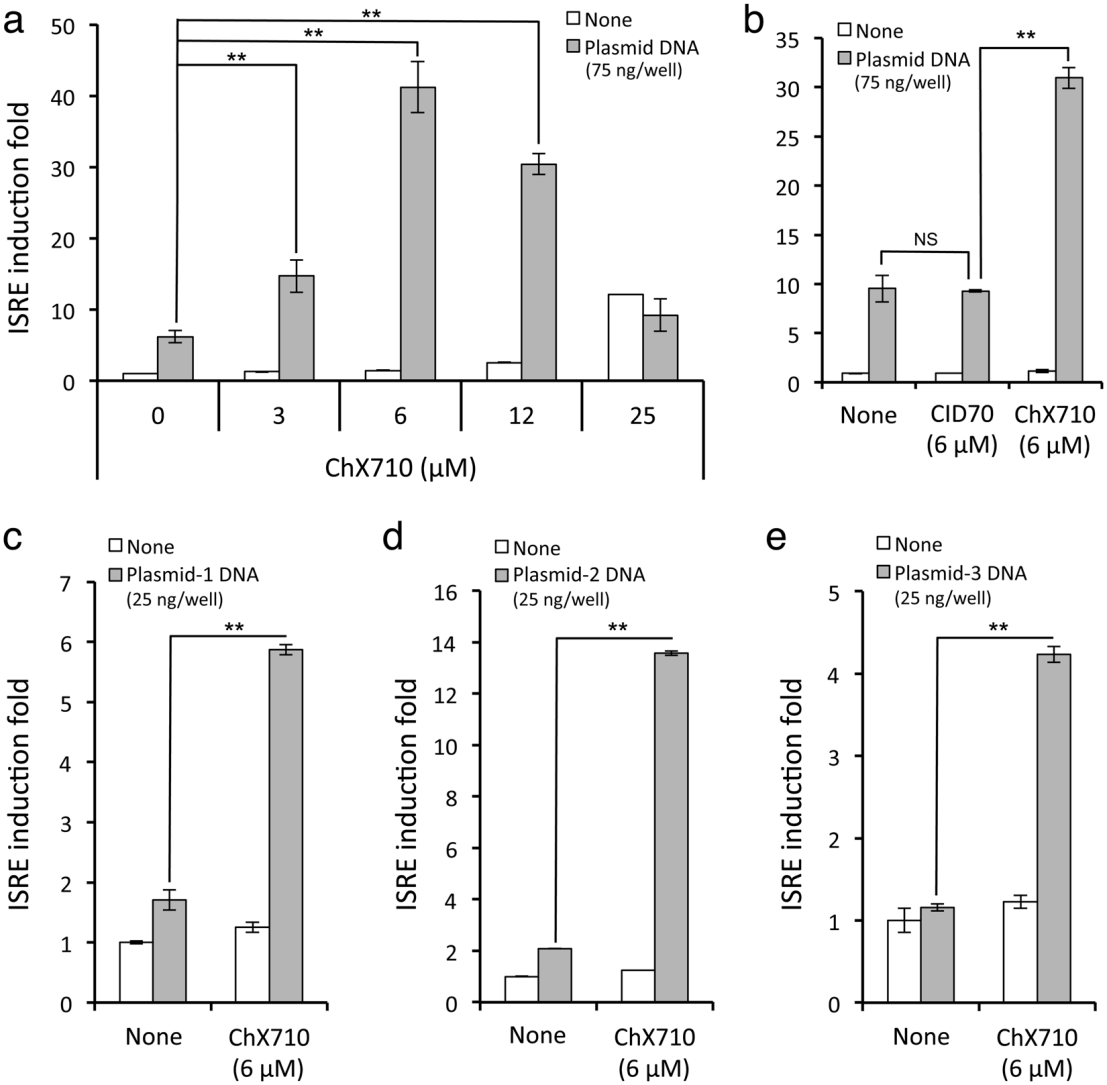
ISRE induction by plasmid DNA transfection is boosted in ChX710-treated cells. (**a**) ISRE-luciferase reporter cells were transfected with 75 ng/well of plasmid DNA (pCiNeo-3xFlag-GW), and cultured at 4 × 10^4^ cells/well in 96-well plates with DMSO alone or increasing concentrations of ChX710. After 24 hours of incubation, luciferase induction was determined. (**b**) Same experiment as in (**a**) but cells were treated with 6 μM of ChX710 or CID70, an inactivate analog of ChX710. (**c**) Same experiment as in (**a**) but cells were transfected with 25 ng/well of pCiNeo-3xFlag-GW (Plasmid-1), pTK-Renilla (Plasmid-2) or pDONR207 (Plasmid-3), and stimulated with ChX710 at 6 μM. Experiments were performed in duplicate, and data represent means ± SD. ***P < 0.01 as calculated by two-way ANOVA and Bonferroni’s post hoc test (**a** and **b**) or Student’s t test (**c**).

Although ChX710 and related compound ChX67779 induced the ISRE-luciferase reporter gene, their capacity to stimulate ISGs is rather limited compared to recombinant IFN-β (Figs 2a,c and 3b,c). We thus determined whether ChX710 could boost the expression of ISGs upon stimulation with suboptimal concentrations of recombinant IFN-α, short synthetic 5′-triphosphate RNA molecules (ssRNA) or plasmid DNA transfection. As shown in Supp. Fig. 5, ChX710 had very limited or no effect on ISRE-luciferase induction by recombinant IFN-α or ssRNA, respectively, but rather suppressed luciferase activity when applied at 25 μM, a phenomenon that is probably linked to the stress response and cytotoxicity associated to this compound. However, ChX710 at 6 μM strongly boosted the cellular response to plasmid DNA transfection as assessed by ISRE-luciferase induction (Fig. 6a). Compared to ChX710, the inactive analog CID70 showed no effect on cellular response to plasmid DNA, supporting the specificity of our observation (Fig. 6b). When reporter cells were transfected with low doses of different plasmids exhibiting various DNA sequences, ChX710 amplified cellular response in all cases (Fig. 6c,d and e). This suggested some unique capacity of ChX710 to amplify cellular response to foreign DNA.

**Figure 7:**
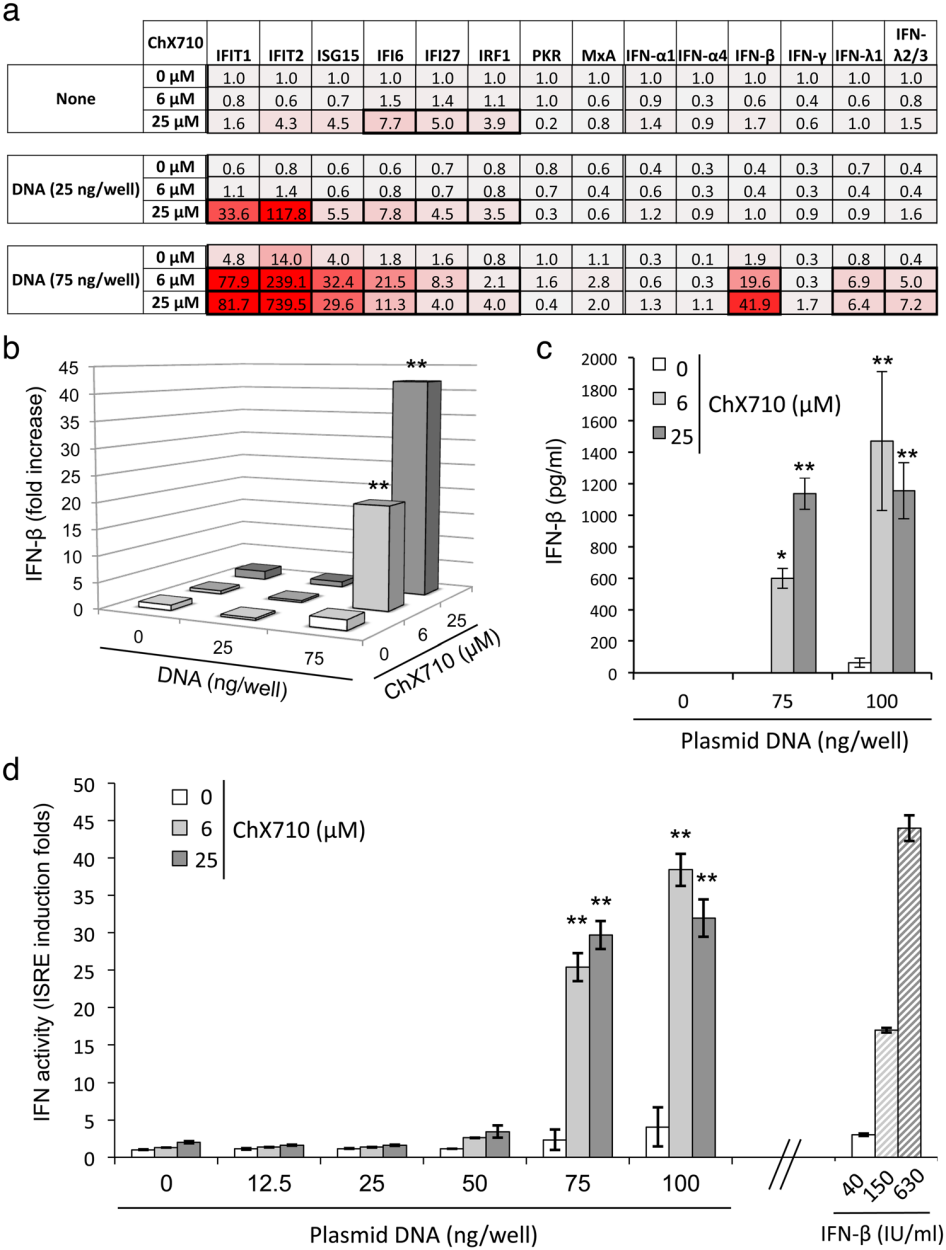
Synergistic induction of ISGs and IFN-β by DNA transfection and ChX710. (**a**) ISRE-luciferase reporter cells were transfected with 25 or 75 ng/well of plasmid DNA (pCiNeo-3xFlag-GW), and cultured at 4 × 10^4^ cells/well in 96-well plates with DMSO alone or ChX710 at 6 or 25 μM. After 24 hours of incubation, total RNA were extracted and expression levels of specified genes were determined by RT-qPCR. Data were normalized with using RPL13A mRNA expression as reference, and expressed as induction folds relative to DMSO-treated cells using the 2^−ΔΔCt^ method. Induction folds >3 that are statistically significant between untreated and ChX710-treated wells are framed with bold lines (two-way ANOVA and Bonferroni’s post hoc test; *P* < 0.05). (**b**) Data corresponding to IFN-β mRNA expression levels as in (A) are presented as 3D histograms to highlight the synergistic effect of DNA transfection and ChX710 treatment. (**c**) HEK-293 cells were transfected with 75 or 100 ng/well of plasmid DNA (pCiNeo-3xFlag-GW), and cultured at 4 × 10^4^ cells/well in 96-well plates with DMSO alone or ChX710 at 6 or 25 μM. After 48 hours of incubation, culture supernatants were recovered, tested for IFN-β concentration by ELISA. (**d**) Same as in (**c**) but culture supernatants were applied at a ½ dilution to fresh ISRE-luciferase reporter cells to determine interferon activity. As a reference, reporter cells were stimulated with recombinant IFN-β at 40, 150 or 630 IU/ml. After 24 hours of incubation, luciferase activity was determined. Data represent means ± SD of three independent experiments. *P < 0.05 and **P < 0.01 correspond to statistically significant differences between untreated and ChX710-treated wells (two-way ANOVA and Bonferroni’s post hoc test).

To further document this observation, we studied a panel of ISGs in HEK-293 cells transfected with plasmid DNA and stimulated with 6 or 25 μM of ChX710 for 24 h (Fig. 7a). First, we confirmed the modest induction of specific ISGs by ChX710 alone. DNA transfection alone was also a poor inducer of ISGs, although a significant expression of specific genes such as IFIT1, IFIT2 or ISG15 was detectable at highest concentrations of plasmid (75 ng/well). In agreement with previous results obtained with the ISRE-luciferase reporter gene, a strong synergistic effect was observed when DNA transfection was combined with ChX710 treatment, a phenomenon restricted to early ISGs and IFN-β. For example, a 14-fold induction of IFIT2 gene was observed when cells were transfected with 75 ng/well of plasmid DNA alone, but a 239-fold induction was detected when ChX710 was added at only 6 μM (Fig. 7a). Quite similarly, ChX710 alone did not induce IFN-β promoter, and the induction observed with 75 ng/well of plasmid DNA was barely detectable in our culture conditions. In contrast, a 42-fold induction was observed when both signals were combined (Fig. 7a and b).

We then determined if this synergism could be confirmed at a protein level by testing culture supernatants for the presence of type I interferon activity. Cells were transfected with plasmid DNA and stimulated with ChX710, and then incubated for 48 hours to allow IFN accumulation in culture medium. Supernatants were recovered and tested for the presence of IFN-β by ELISA (Fig. 7c), or diluted twice and applied to fresh ISRE-luciferase reporter cells for the detection of interferon activity. As shown in Fig. 7c, high levels of IFN-β were detected in culture supernatants when cells were transfected with plasmid DNA and co-treated with ChX710. The corresponding interferon activity was also detected in culture supernatants when applied to ISRE-luciferase reporter cells (Fig. 7d). Detected interferon activity was comparable to >100 IU/ml of recombinant IFN-β, thus demonstrating high levels of interferon secretion. We noticed that ChX710 at 25 μM did not perform much better than a lower concentration of 6 μM despite stronger synergistic effects on IFN-β mRNA levels (Fig. 7a and b). This probably relates to the significant cellular cytotoxicity and stress associated to 25 μM of ChX710.

### Synergistic ISRE activation by plasmid DNA and ChX710 is STING dependent

**Figure 8:**
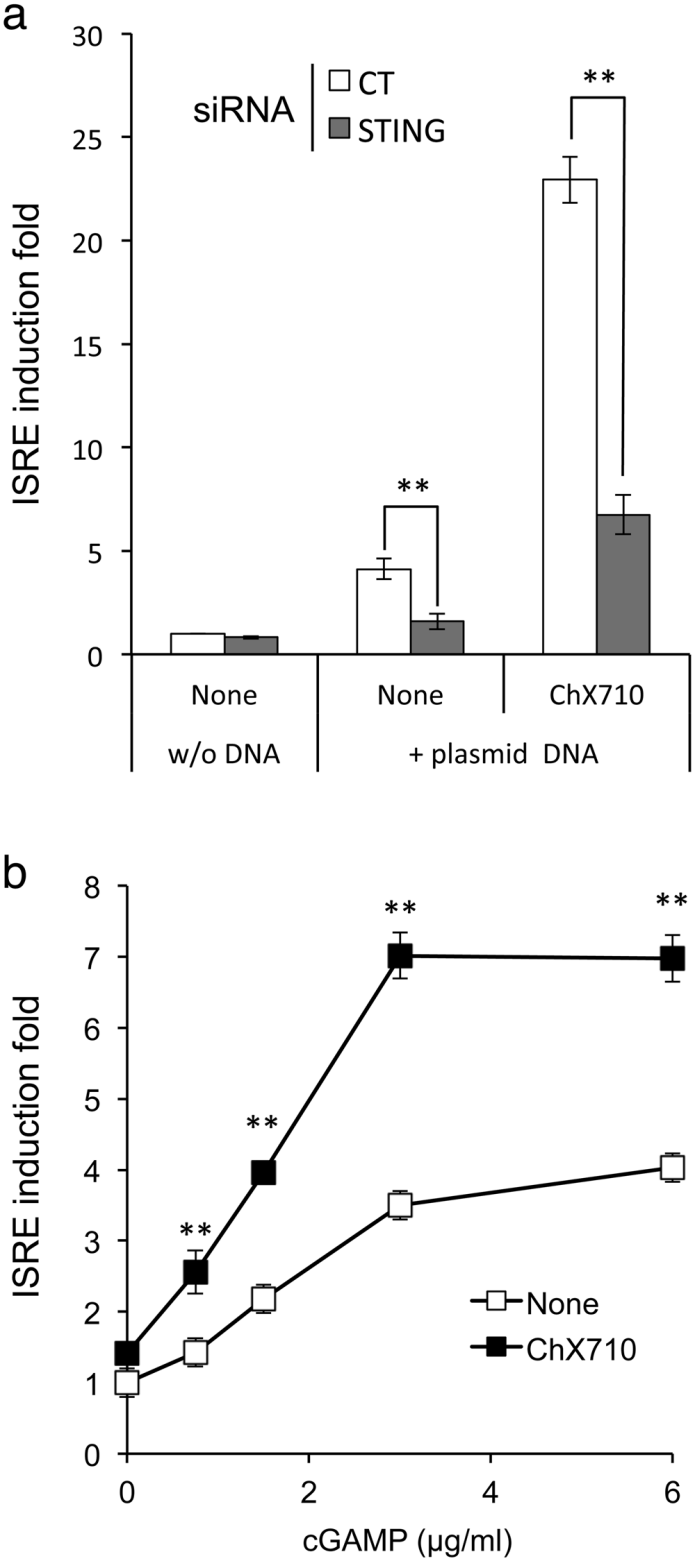
Synergistic ISRE induction by DNA transfection and ChX710 is STING dependent. (**a**) STING was silenced by siRNA transfection in ISRE-luciferase reporter cells. After 48 hours of cultures, cells were transfected with plasmid DNA (pCiNeo-3xFlag-GW) and stimulated with ChX710. After 24 hours of incubation, luciferase induction was determined. Data represent means ± SD of three independent experiments. (**b**) ISRE-luciferase reporter cells were treated with 2′,3′-cGAMP at indicated concentrations with ChX710 (6 μM) or DMSO alone. After 24 hours of incubation, luciferase induction was determined. Experiment was performed in duplicate, and data represent means ± SD. **P < 0.01 corresponds to statistically significant differences as calculated by two-way ANOVA and Bonferroni’s post hoc test.

Cellular adaptor STING is known for its critical role in cytosolic DNA sensing and subsequent induction of innate immunity in many systems. To investigate the role of STING in the cellular response to concomitant plasmid DNA transfection and ChX710 treatment, expression of this protein was inhibited by siRNA. ISRE-luciferase reporter cells were transfected with STING-specific siRNA then transfected after 48 hours with plasmid DNA and treated or not with ChX710. As assessed by luciferase expression levels, ISRE induction by DNA transfection alone was STING dependent in agreement with previous reports (Fig. 8a). When cells were both transfected with DNA and treated with ChX710, ISRE induction was deeply inhibited by STING depletion (Fig. 8a). These results demonstrated that ChX710 primes DNA sensing and the interferon response along a signaling cascade that relies on the adaptor protein STING. We thus determined if ChX710 could also amplify the cellular response to 2′,3′-cGAMP, which is synthesized by cGAS upon DNA sensing and directly activates STING. As shown in Fig. 8b, ChX710 enhanced ISRE-luciferase induction by 2′,3′-cGAMP, thus establishing that signaling events at the level or downstream of STING are modulated by this compound.

## Discussion

Thanks to the functional screening of a chemical library, we have identified ChX710 from a series of 1H-benzimidazole-4-carboxamide derivatives that boost the type I IFN response and cellular sensing of transfected DNA. This compound activates the ISRE promoter sequence through a signaling axis involving MAVS and IRF1, and its immunostimulatory properties were confirmed by showing some modest but significant induction of specific ISGs. Interestingly, ISG induction profiles were cell-type dependent. This could be explained by different expression levels of the multiple factors controlling ISGs such as ubiquitin or SUMO ligases for example^36^, variations in the epigenetic landscape and the accessibility of ISGs to the transcriptional machinery^37, 38^, or some resistance to the functional stress induced by this molecule, as suggested by the lower cytotoxicity and the lack of ISGs induced in MRC5. We also showed IRF3 phosphorylation at Ser386 in ChX710-treated cells, probably in association with other post-translational modifications that need to be characterized (Fig. 5e and f). However, levels of IRF3 phosphorylation or phosphorylated residues were probably inadequate to significantly induce IFN-β expression in a context where NF-κB also remained inactive (Fig. 1c), thus precluding the assembly of a complete IFN-β enhanceosome. Nevertheless, ChX710 treatment was found to efficiently prime the cellular response to DNA transfection *via* STING as assessed by strong synergistic effects on IFN-β expression at both transcriptional and protein levels. This finding strongly supports the emerging idea that the type I IFN response to nucleic acids can be boosted using *ad hoc* molecules, opening new perspectives in anti-infectious and cancer therapies.

The mode of action of ChX710 remains undetermined and multiple hypotheses could explain its biological properties. Nevertheless, and to our knowledge, it is the first time that a biological activity is associated to the ISRE-inducing chemical series enlisted in Supp. Table 1. To get insight into the mode of action of ChX710 and related active compounds, we searched chemical databases such as PubChem, Hitpick, Reaxys or Scifinder to identify chemical analogues with already known targets^39–41^. Some 1H-benzimidazole-4-carboxamide derivatives are well-characterized inhibitors of Poly (ADP-Ribose) Polymerase (PARP), a nuclear enzyme family essentially involved in the detection and signal of single-strand DNA damages to the DNA repair machinery^42^. Other compounds structurally related to ChX710 chemical series were also reported for their antiviral activity against enteroviruses, but their mode of action is unknown^43^. Future investigations are required to explore potential links between these different observations. Another lead that could help decipher the mode of action of ChX710 comes from the MAVS/IRF1 signaling axis that is involved in ISRE-luciferase activation by this molecule. Indeed, some important crosstalk between MAVS and STING have been reported^44^. When signaling through MAVS, ChX710 could therefore influence DNA sensing and signaling events at the level of STING. Quite interestingly, both MAVS and IRF1 have been also involved in the sensing of viruses by RIG-like receptors at the membrane of peroxisomes^45^. Peroxisomes are cytoplasmic organelles delimited by a membrane bilayer that use O_2_ to oxidize a variety of molecules, including toxic molecules for neutralization, and produce H_2_O_2_ that is further used by catalases for oxidation. In addition, they were recently characterized as a signaling platform critically involved in activation of the interferon response. Finally, it has been recently shown that UV light or chemical carcinogens such as benzo[a]pyrene-7,8-dihydrodiol-9,10-epoxide or N-acetyoxy-2-acetylaminofluorene prime the cellular response to cytosolic DNA^46^. This cellular response involves the activation of caspases as a consequence of DNA damages, and subsequent degradation of the AMBRA1/ULK1 complex. Since this complex is involved in the negative regulation of STING, its degradation enhances the interferon response to cytosolic DNA. In contrast, cellular response to 5′-triphosphate RNA is not increased, which parallels our observations. Interestingly, we found that ChX710 treatment was associated to the loss of ULK1 in HEK-293 cells (Supp. Fig. 6). This suggests ChX710 downregulates the expression of STING inhibitors such as the AMBRA1/ULK1 complex, which would account for an amplified interferon response to cytosolic DNA.

Cytosolic DNA sensing is playing an important role in the immune response to DNA viruses and intracellular bacteria, but also against tumor cells^4, 5^. Indeed, DNA damages and rapid cellular cycling are associated to the leakage of DNA replication byproducts from the nucleus to the cytoplasm, which can then prime the STING signaling cascade^47^. In some cases, mitochondrial stress can also lead to STING activation by mitochondrial DNA release in the cytosol^48^. Therefore, drugs that enhance the cellular response to cytosolic DNA could be of great interest in the treatment of various infections and cancer therapy. Indeed, this could boost the immune response by increasing type I IFN secretion, and sensitizing infected or tumor cell to apoptosis. In cancer treatment, enhancers of cytosolic DNA sensing could be combined with existing chemotherapies inducing DNA damages to block cellular proliferation such as cisplatin or etoposide. Further investigations are needed to determine if ChX710 is a potential lead molecule for these applications, although the intrinsic cytotoxicity of this molecule should be taken into account and could be a limitation depending on target cells. But more importantly at this point, our work is supporting the idea that cytosolic DNA sensing can be chemically enhanced and in the near future, we would like to use ChX710 as a molecular probe to identify the signaling pathways involved.

## Methods

### Chemical library and compounds

All compounds were purchased from CHEM-X-INFINITY (Romainville; www.chem-x-infinity.com). The initial compound collection amounts to a total of 10,000 molecules arrayed in 125 96-well microplates. Twenty-three chemical families are represented in this library, and compounds have molecular weights ranging from 250 to 550 g/mol. All compounds were stored in DMSO at −20 °C at 10 mM. Following the screen of this library, selected hits and derivatives were obtained from CHEM-X-INFINITY. As assessed by the manufacturer, purity levels were >90% for all compounds, whereas ChX67779 and ChX710 were pure at 99% and 98%, respectively. 2′,3′-cGAMP was from Sigma-Aldrich (SML1229).

### Cell lines, culture medium and luciferase assays

All cells were maintained in Dulbecco’s modified Eagle’s medium (DMEM; Gibco-Invitrogen) containing 10% fetal calf serum (FCS), penicillin, and streptomycin at 37 °C and 5% CO_2_. A549, MRC5 and Vero cells were from ATCC. The reporter cell line “STING-37” was previously described^29^. Briefly, the ISRE-luciferase reporter gene was amplified by PCR from pISRE-luciferase plasmid (Stratagene, Ref 219089), and inserted in a vector with a G418-resistance gene. This new plasmid was transfected in HEK-293 cells from ATCC, amplified in culture medium supplemented with G418 at 500 μg/ml, and cloned by serial dilution. A total of 44 individual clones were screened, and STING-37 clone was selected for the optimal luciferase induction detected upon stimulation with recombinant IFN-β.

Cellular viability was determined by quantification of adenosine triphosphate (ATP) in culture wells using the CellTiter-Glo Assay (Promega) following manufacturer’s recommendations. Luciferase induction in “STING-37” cells was determined by addition of 50 µl/well of Bright-GLO reagent (Promega) or Britelite plus reagent (PerkinElmer), and measured during 0.1 s with a luminometer (Enspire; PerkinElmer).

### Screening procedure

The screening procedure was performed as following with a TECAN Freedom EVO platform. Compounds from mother plates were diluted in DMSO at 2 mM to obtain intermediate dilution plates. Then, compounds were transferred into white, flat bottom, bar-coded tissue culture 96-wells plates (Greiner Bio One): 1 μl of a DMSO solution was spiked into dry wells of daughter plates (80 compounds per plate). For each plate, columns 1 and 12 were dedicated to controls: culture wells were alternatively spiked with 1 µl of DMSO alone (negative control) or supplemented with recombinant IFN-β so that final concentration equals 1,000 IU/ml (positive control). Finally, 100 μl of a STING-37 cell suspension at 4.10^5^ cells/ml was added to each well of the microplate already containing one chemical compound (final concentration was 20 μM). After 24 hours of incubation at 37 °C in the presence of 5% CO_2_, the firefly luciferase substrate (Bright-Glo, Promega) was added directly into the wells (50 µl) and luciferase activity was measured 6 minutes later on a Infinite® M1000 Pro (TECAN) using a 0.1 s integration time. For each plate, means of luminescence and corresponding standard deviations were calculated for positive and negative controls (μ^+^, σ^+^, μ^−^, and σ^−^, respectively) to determine the signal-to-background ratio (S/B = μ^+^/μ^−^) and the Z’-factor (Z’-factor = 1–3*(σ^+^ + σ^−^)/(μ^+^−μ^−^)). Average Z’-factor was determined to be 0.85 ± 0.06 (no value below 0.62) and signal-to-background (S/B) ratio, which corresponds to luciferase signal in the presence of recombinant IFN-β relative to DMSO alone, was on average average 77 ± 24 for all plates, and always >26. Altogether, this demonstrated the robustness of our assay, which can be categorized as excellent^49^. For each compound, the induction factor was calculated as the ratio of luminescence signal measured in the corresponding well to the mean of luminescence for negative controls in the same plate.

### Reagents and plasmids

The pISRE-Luc and pNF-κB-Luc reporter plasmids were from Stratagene (Ref 219089 and 219078, respectively). Recombinant IFN-β was from PBL Assay Science (Ref 11410-2). Recombinant TNF-α was from R&D Systems (Ref 210-TA). Short synthetic 5′-triphosphate RNA molecules (ssRNA) were synthesized from pCI-neo vector digested with XbaI using T7 RiboMAX Express large scale RNA production system (Promega), and then purified with a filtering membrane (Millipore). Sheep polyclonal antibodies against IFN-α (31100-1) and IFN-β (31400-1) were from PBL Assay Science. The VeriKine Human IFN-β ELISA kit was from PBL Assay Science. DNA or RNA transfections were performed with JetPrime PEI following manufacturer’s recommendations (Polyplus transfection). The following plasmids were transfected to activate ISRE promoter sequences: pCiNeo-3xFlag-GW, pTK-Renilla (Promega), pDONR207 (ThermoFisher).

### Quantitative RT-PCR analysis

Cells were plated in 24-well plates (2 × 10^5^ cells per well), treated the same day with ChX67779 or ChX0306710 (ChX710), and transfected with plasmid DNA when specified using JetPrime reagent. One day later, cells were recovered in PBS and total RNA isolated with the RNeasy Mini Kit (Qiagen) according to manufacturer’s protocol. Following elution, RNA yields were evaluated using a Nanodrop spectrophotometer (Nanodrop technologies). Samples were analyzed by capillary electrophoresis (Agilent) to verify the absence of RNA degradation.

To measure transcription levels of the 11 ISGs presented in Fig. 2a, a two-step qRT-PCR (Taqman technology, Applied Biosystems) was performed using commercial primers from ThermoFisher Scientific: *IFI27* (Hs00271467_m1), *IFI35* (Hs00413458_m1), *IFI44* (Hs00197427_m1), *IFI6* (Hs00242571_m1), *IFIH1* (Hs01070332_m1), *IFIT1* (Hs01911452_s1), *IFIT3* (Hs01922752_s1), *IFITM1* (Hs00705137_s1), *ISG15* (Hs01921425_s1) and *MX1* (Hs00895608_m1). Expression levels of four housekeeping genes, including *18S* (Hs99999901_s1), *GAPDH* (Hs99999905_m1), *GUSB* (Hs99999908_m1), and *HPRT1* (Hs99999909_m1), were also determined and used as internal reference controls. Starting from 1 μg of total RNA, cDNA synthesis was achieved in 20 μL using the SuperScript VILO cDNA Synthesis Kit following manufacturer’s recommendations (Life Technologies). Quantitative PCR reactions were performed on 0.6 μL of cDNA synthesis reaction mix using the TaqMan Fast Advanced Master Mix (Applied Biosystems) on a StepOnePlus™ Real-Time PCR machine (Applied Biosystems). Transcripts were quantified using the following program: 20 s at 95 °C followed by 40 cycles of 1 s at 95 °C and 20 sec at 60 °C. Results were normalized using expression levels of the four housekeeping genes.

Transcription levels of type I IFN genes and ISGs presented in Fig. 7 were determined by RT-qPCR using the following protocol. RNA samples were converted to cDNA with RevertAid H Minus First Strand cDNA Synthesis Kit (Thermo Scientific). Real-time PCR reactions were performed in duplicates using Takyon ROX SYBR MasterMix blue dTTP (Eurogentec) on a 7900HT Fast Real-Time PCR System (Applied Biosystems). Transcripts were quantified using the following program: 3 min at 95 °C followed by 35 cycles of 15 s at 95 °C, 25 s at 60 °C and 25 s at 72 °C. Values for each transcript were normalized to expression levels of RPL13A (60S ribosomal protein L13a) using the 2^−ΔΔCt^ method. Primers used for quantification of transcripts by real time quantitative PCR are indicated in Supp. Table 3.

### Isolation and culture of human peripheral blood mononuclear cells (PBMCs)

Blood from healthy blood bank donors was obtained from *“Etablissement Français du Sang”* (Convention # 07/CABANEL/106; Paris; France). Human peripheral blood mononuclear cells (PBMC) were isolated by density centrifugation with Lymphoprep medium (StemCell Technologies), and cultured in RPMI-1640 (Sigma-Aldrich; R8758) containing 10% fetal bovine serum.

### siRNA procedure

Silencer Select siRNA were purchased from Invitrogen, and transfected in STING-37 cells following manufacturer’s recommendations. Gene silencing was achieved with pools of two siRNA for STAT1 (s278/s277), STAT2 (s13530/s13529), MAVS (s33178/s33180), STING (s50646/s226307), IRF1 (s7502/s7503), IRF3 (s7507/s7509), whereas controls correspond to a pool of two siRNA directed against IRF5 (s7513 and s7515). In each well of a 96-well plate, 3 pmol of siRNA were mixed with 20 μL of Opti-MEM (Gibco-Invitrogen) and 0.25 μL of Lipofectamine RNAiMAX transfection reagent (Invitrogen). This mix was incubated for 10 minutes at room temperature, and supplemented with 80 μL of DMEM +10% FCS without penicillin and streptomycin and 15,000 STING-37 cells. Cells were incubated for 48 hours at 37 °C and 5% CO_2_, and then stimulated with ChX710 or recombinant IFN-β, or transfected with ssRNA or plasmid DNA using JetPrime reagent. After 24 hours of culture, firefly luciferase activity was determined.

### Western blot analysis

Briefly, 2 × 10^5^ HEK-293T or A549 cells were dispensed in each well of a 24-well plate with ChX710 or DMSO alone. After 24 hours of culture, cells were washed in PBS and resuspended in RIPA lysis buffer (1% Nonidet P-40, 0.5% Na-deoxycholate, 0.1% SDS, 50 mM Tris–HCl at pH 7.4, 150 mM NaCl, 2 mM EDTA, and 50 mM NaF) supplemented with Complete Protease Inhibitor Cocktail (Roche). After incubation on ice for 20 min, cell lysates were clarified by centrifugation at 14,000 × g for 10 min. Protein extracts were resolved by SDS-polyacrylamide gel electrophoresis (SDS-PAGE) on 4–12% NuPAGE Bis–Tris gels with MOPS running buffer (ThermoFisher), and transferred to a nitrocellulose membrane. Proteins were detected using standard immunoblotting techniques using the following primary antibodies: anti-IRF1 rabbit monoclonal antibody was from Cell Signaling (Clone D5E4; Ref. 8478S), anti-IRF3 rabbit monoclonal antibody was from Cell Signaling (Clone D6I4C; Ref. 11904), anti-P-IRF3 (Ser386) rabbit polyclonal antibody was from Millipore (ABE501), anti-STAT1 mouse monoclonal antibody was from Cell Signaling (Clone 9H2; Ref. 9176), anti-STAT2 rabbit polyclonal antibody was from Cell Signaling (Ref. 4594), anti-MAVS rabbit polyclonal antibody was from Alexis (Ref AT107; 210–929-C100), anti-STING rabbit monoclonal antibody was from Cell Signaling (Clone D2P2F; Ref 13647), anti-ULK1 rabbit monoclonal antibody was from Cell Signaling (Clone D8H5; Ref 8054), and anti-β-Actin mouse monoclonal antibody was from Sigma-Aldrich (Clone AC-15; Ref. A5441). Secondary anti-mouse and anti-rabbit HRP-conjugated antibodies were from GE-Healthcare. Protein detection was performed using the SuperSignal West Pico Chemiluminescent Substrate (ThermoFisher Scientific).

## Electronic supplementary material

Supplementary Information
