# Supplementary material for: Identification of a small molecule that primes the type I interferon response to cytosolic DNA
Source: Sci Rep. 2017 May 31;7:2561. doi: 10.1038/s41598-017-02776-z (PMC5451460; doi:10.1038/s41598-017-02776-z)
Supplement: Supplementary file 1 — Supplementary Information [file 41598_2017_2776_MOESM1_ESM.pdf]

SUPPLEMENTARY INFORMATION

Identification of a small molecule that primes the type I interferon response to cytosolic DNA

Samira Khiar[1], Marianne Lucas-Hourani[1], Sébastien Nisole[2], Nikaïa Smith[3], Olivier Helynck[4], Maryline Bourgine[5], Claude Ruffié[1], Jean-Philippe Herbeuval[3], Hélène Munier-Lehmann[4], Frédéric Tangy[1], Pierre-Olivier Vidalain[3]

Supplementary Figure S1. Viability of HEK-293T, A549, MRC5, and Vero cells when treated with ChX79. Cells were incubated with indicated concentrations of ChX79. After 24 hours of culture, the number of living cells was determined by ATP quantification using the CellTiter-GLO reagent. Results are expressed as a percentage relative to DMSO-treated cells. ** $P<0.01$ corresponds to statistically significant differences between cell types as calculated by two-way ANOVA and Bonferroni's post hoc test.

Supplementary Figure S2. Cellular toxicity of ChX710 and analog compound CID70. HEK-293 cells with the ISRE-luciferase reporter gene (STING-37 cell line) were incubated with increasing concentrations of CID70 **(a)** or ChX710 **(b)**. After 0, 24 and 48 hours of culture, the number of living cells was determined using the CellTiter-GLO reagent. Results are expressed as a percentage relative to the initial number of living cells at t=0 hours. ** $P<0.01$ as calculated by one-way ANOVA with Tukey's post hoc test. **(c)** Human PBMCs from two independent donors were treated with increasing concentrations of ChX710. After 24 hours of culture, the number of living cells was determined using the CellTiter-GLO reagent. Results are expressed as a percentage relative to DMSO-treated cells.

Supplementary Figure S3. STAT2, but not STAT1, is essential to ISRE activation by IFN-β. (a) STAT1, STAT2 or STAT1 and STAT2 were silenced by siRNA transfection in ISRE-luciferase reporter cells. After 48 hours of cultures, STAT1 and STAT2 expression levels were determined by western-blot analysis. STAT2 expected size is 113 kDa. * indicates bands possibly corresponding to STAT2 degradation intermediates. **(b)** In parallel, cells were stimulated with recombinant IFN-β. After 24 hours of incubation, luciferase induction was determined. Data represent means ± SD of four independent experiments. ** $P < 0.01$

corresponds to statistically significant differences as calculated by two-way ANOVA and Bonferroni's post hoc test.

Supplementary Figure S4. Functional validation of siRNA targeting MAVS, STING, IRF1 and IRF3. (a) The silencing of STING, MAVS, IRF1 and IRF3 by siRNA was validated by western-blot **(b)** MAVS was silenced by siRNA transfection in ISRE-luciferase reporter cells. After 48 hours of cultures, cells were left untreated or transfected with ssRNA. After 24 hours of incubation, luciferase induction was determined. **(c)** Same experiment as (b) using STING-specific siRNA and cGAMP stimulation. **(d)** Same experiment as (b) using IRF1 or IRF3-specific siRNA. **(e)** IRF3 was silenced by siRNA transfection in HEK-293T cells. After 48 hours of culture, cells were transfected with pIFN-β-Luciferase reporter plasmid alone or ssRNA. After 24 hours of incubation, luciferase induction was determined. **(f)** Schematic model of the signaling pathways induced by ssRNA in HEK-293 cells. Experiments were performed in triplicate and data represent means ± SD. ** $P<0.01$ as calculated by Student's t test.

Supplementary Figure S5. Impact of ChX710 on ISRE induction by recombinant IFN-α or ssRNA. (a) ISRE-luciferase reporter cells were treated with both recombinant IFN-α and ChX710 at different concentrations. After 24 hours of incubation, luciferase induction was determined. **(b)** Same experiment as in (a) but cells were treated with different concentrations of ChX710 and transfected with ssRNA. Experiments were performed in duplicate and data represent means ± SD. ** $P < 0.01$ corresponds to statistically significant differences as calculated by two-way ANOVA and Bonferroni's post hoc test.

Supplementary Figure S6. ULK1 degradation is induced by ChX710. HEK-293 cells were treated for 24 hours with DMSO alone or increasing concentrations of ChX710. ULK1 expression was determined by western-blot analysis on protein extracts. ULK1 expected size is 120 kDa. * indicates bands possibly corresponding to ULK1 degradation intermediates.

Supplementary Figure 1

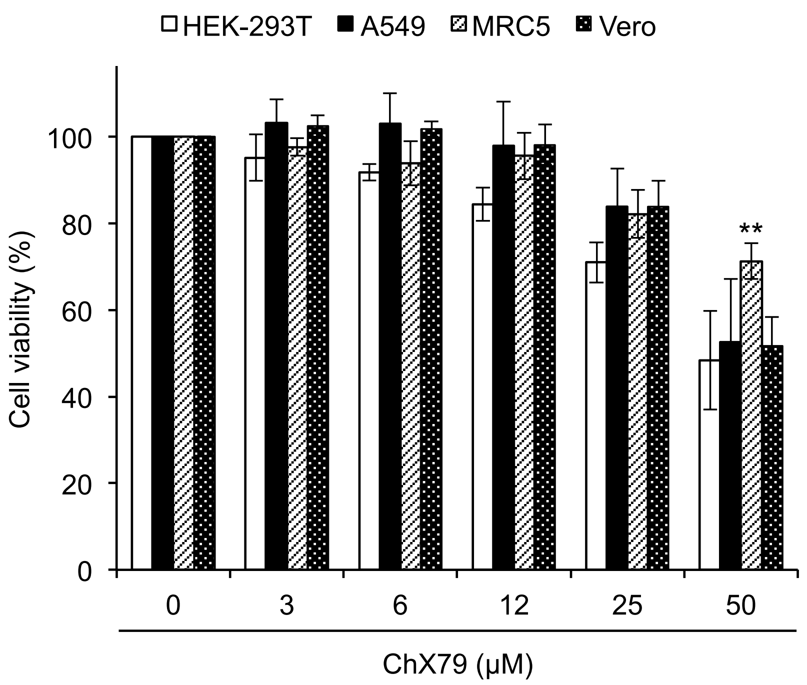

Supplementary Figure 2

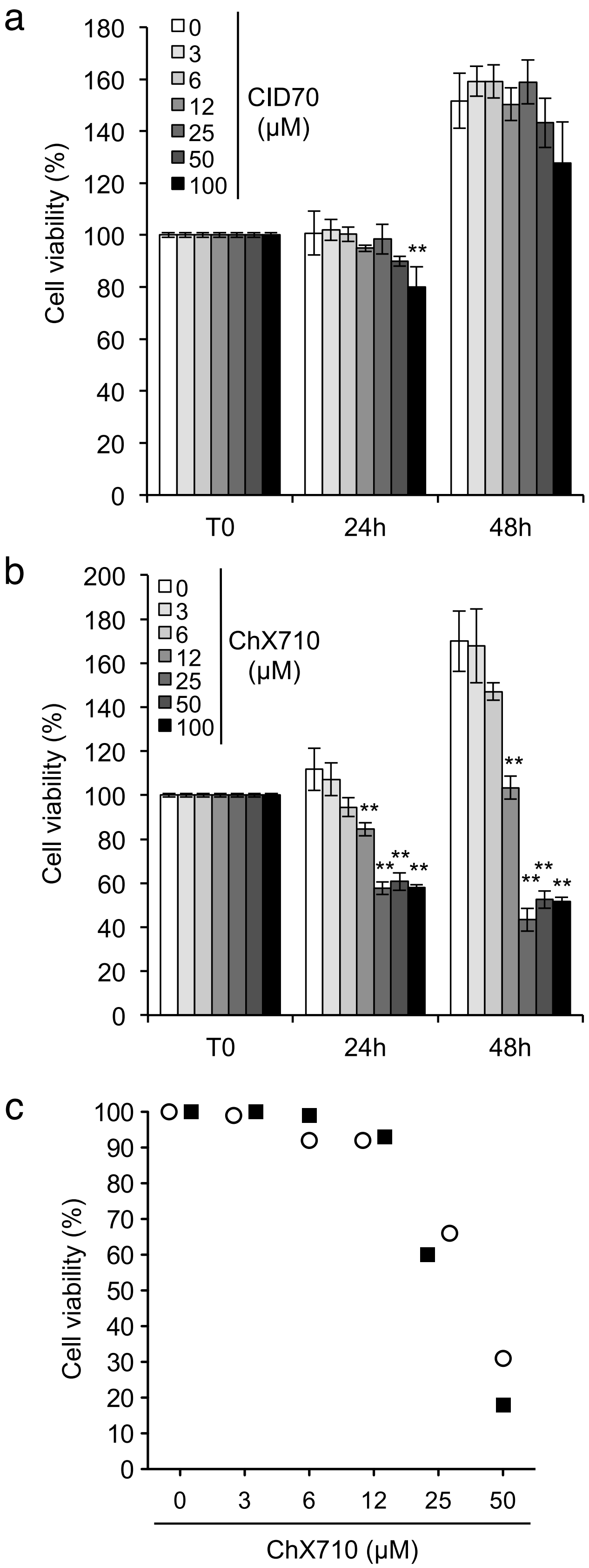

Supplementary Figure 3

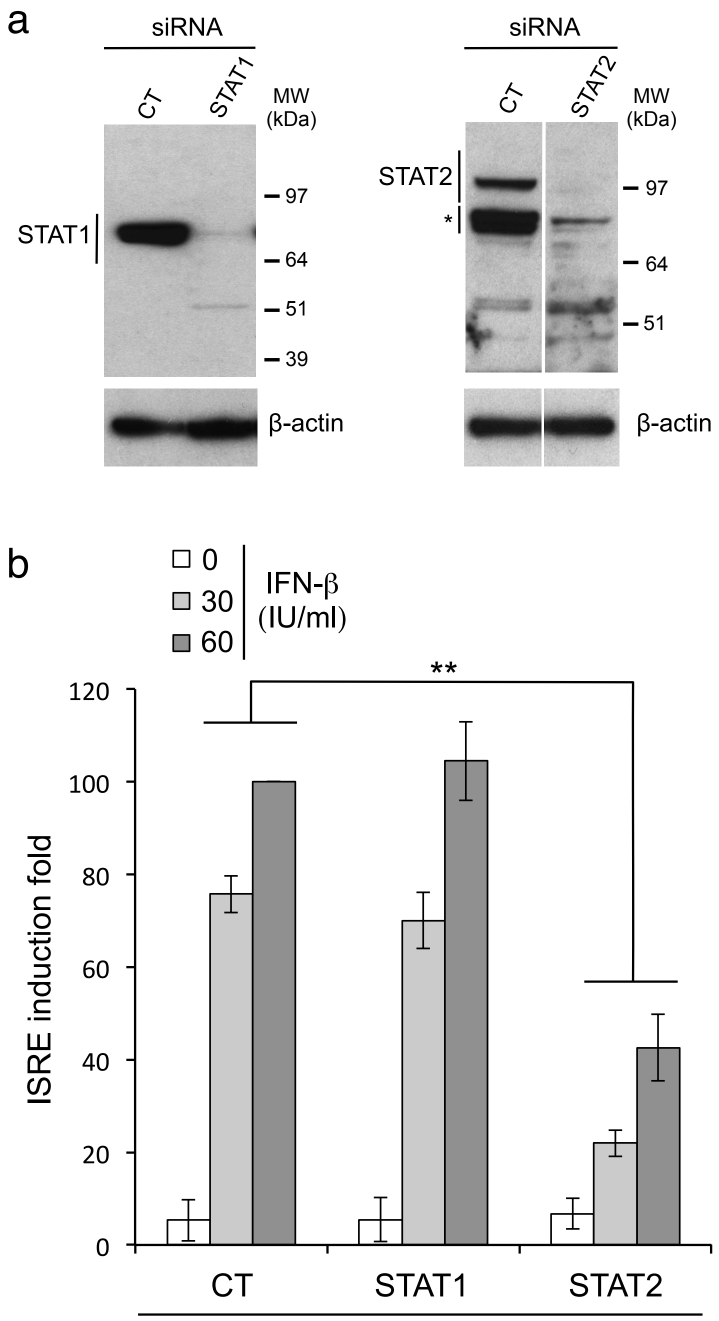

Supplementary Figure 4

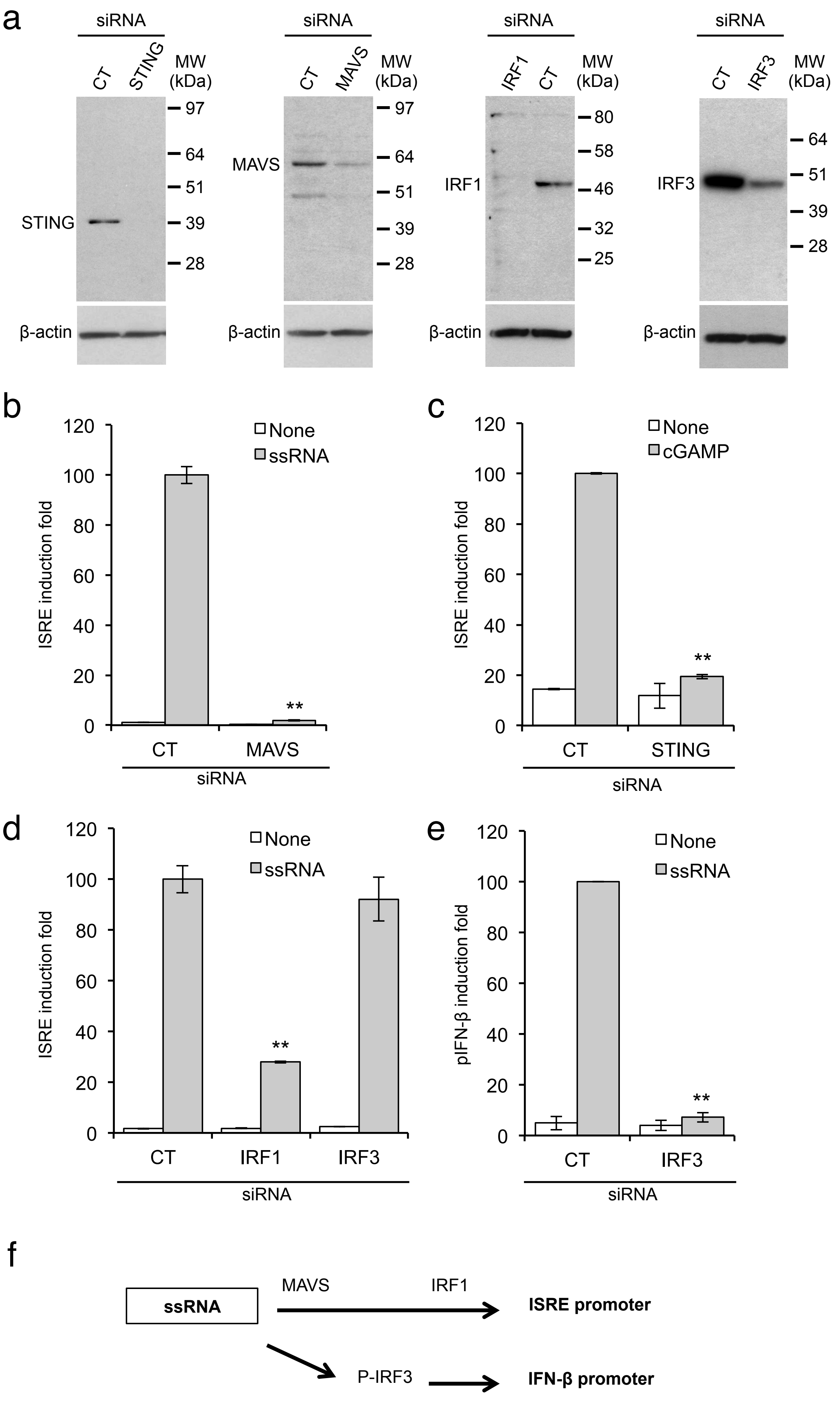

Supplementary Figure 5

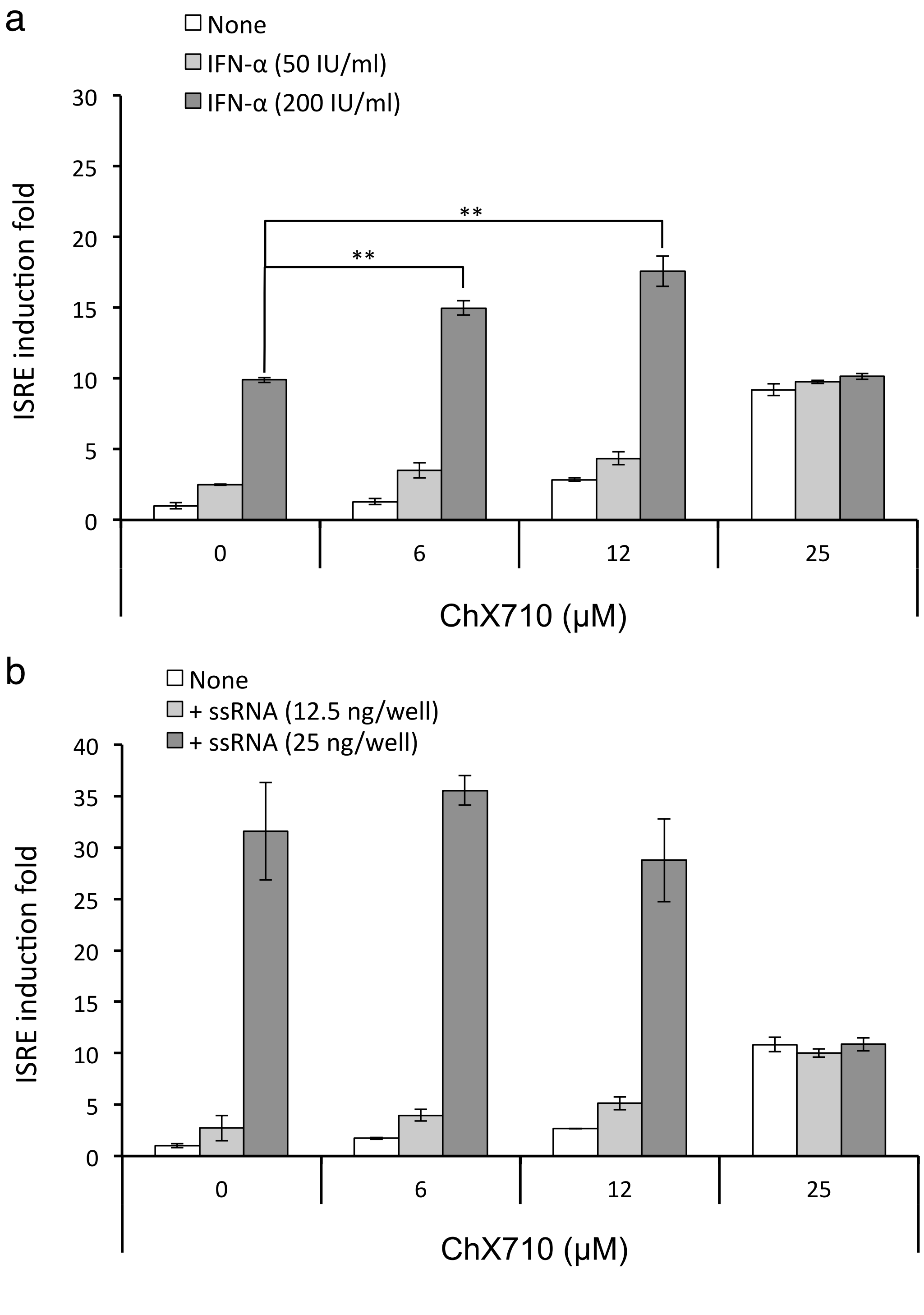

Supplementary Figure 6

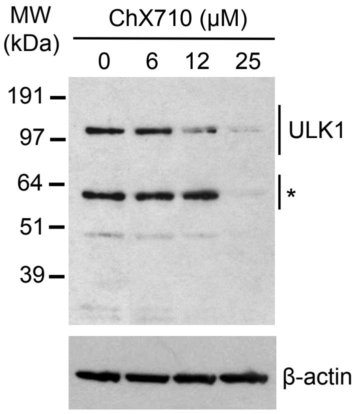

Supplementary Table S1. Chemical structures of ChX67779 analogs and corresponding ISRE-luciferase induction levels. Hits from the initial screen are highlighted in yellow color. Compound concentrations inducing the ISRE-luciferase by >2-folds are in pink color.

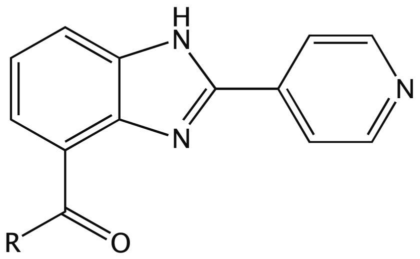

Compound ID	R	ISRE induction fold		
		12 μM	25 μM	50 μM
**ChX67779**	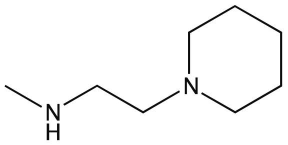	1.6	5.0	9.0
**ChX0275199**	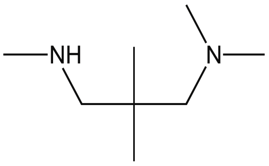	2.6	3.4	3.0
**CID11207170 (CID70)**	—OH	1.0	0.9	0.8
**ChX0306710 (ChX710)**	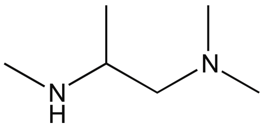	3.8	17.0	0.5
**ChX0306715**	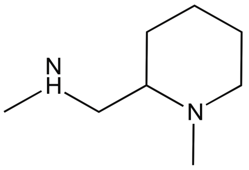	4.2	9.4	1.6

**ChX0306701**	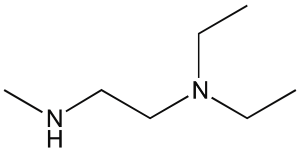	1.4	2.4	10.2
**ChX0306718**	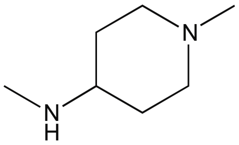	1.6	3.0	6.7
**ChX0306689**	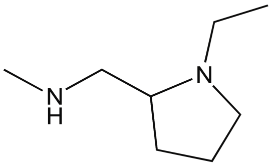	1.8	4.6	3.2
**ChX0306714**	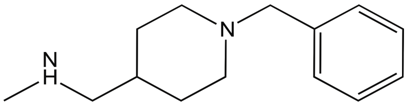	1.1	1.2	7.8
**ChX0306709**	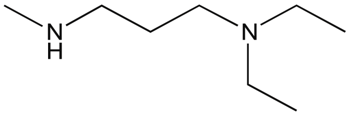	1.5	1.6	4.6
**ChX0306681**	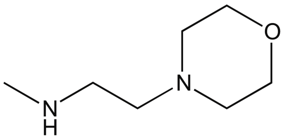	0.9	0.9	0.9
**ChX0306675**	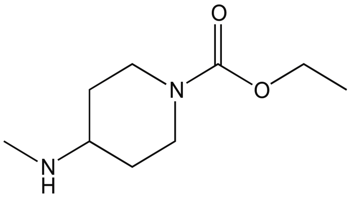	1.0	1.0	0.9
**ChX0274736**	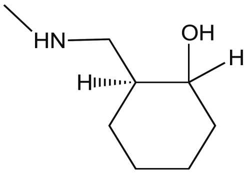	1 .1	1 .1	1 .1

**ChX0275179**	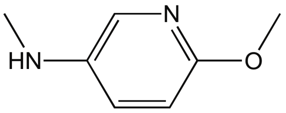	1.2	1.4	1.4
**ChX0306698**	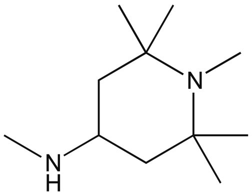	1.4	1.4	1.8
**ChX0306688**	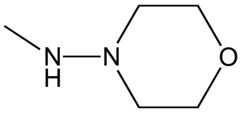	0.8	0.9	1.0
**ChX0275183**	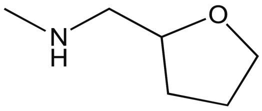	1.1	1.2	1.1
**ChX0275193**	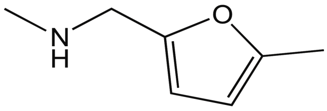	1.0	0 .8	0.5
**ChX0275191**	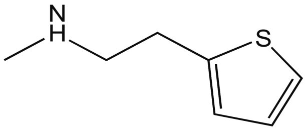	1.2	1.4	1.3
**ChX0275204**	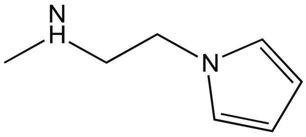	0.9	0.7	0.6
**ChX0306682**	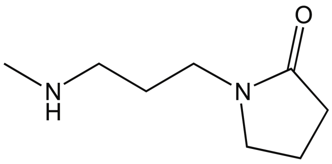	0.9	0.9	0.8

**ChX0306685**	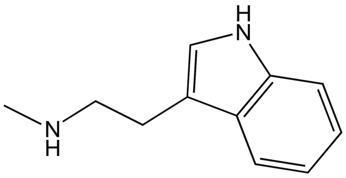	0.4	0.5	0.6
**ChX0276286**	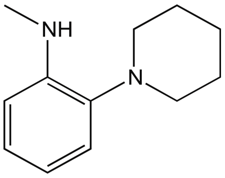	1.2	1.2	1.2
**ChX0306700**	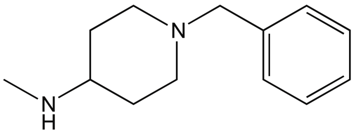	1.1	1.3	1.1
**ChX0306673**	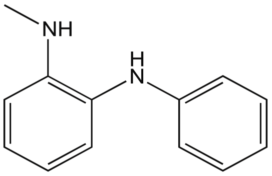	0.9	0.7	0.2
**ChX0306702**	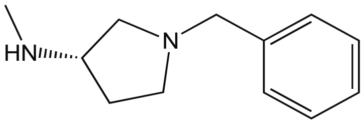	0.9	0.8	0.2
**ChX0306680**	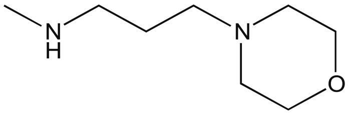	0.9	0.9	0.9
**ChX0306712**	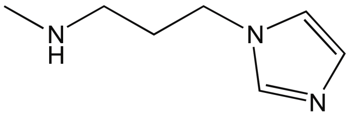	1.0	0.8	0.8
**ChX0306716**	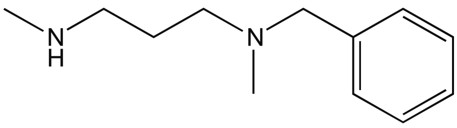	0.9	0.9	0.7

**ChX0306679**	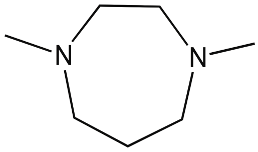	1.0	0.9	0.8
**ChX0306684**	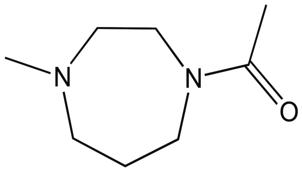	1.0	0.7	0.8
**ChX0306694**	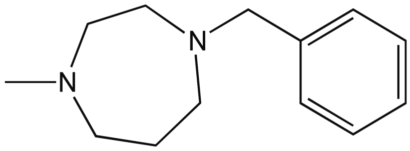	0.7	0.9	0.9
**ChX0306704**	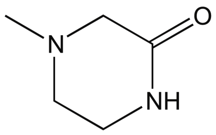	1.0	0.8	0.8
**ChX0306705**	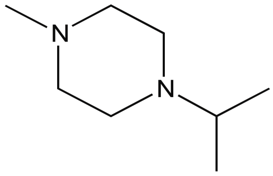	0.8	1.0	0.9
**ChX0306677**	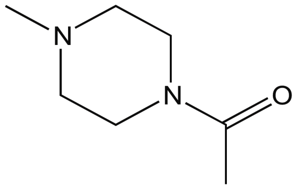	0.9	0.7	0.8
**ChX0306686**	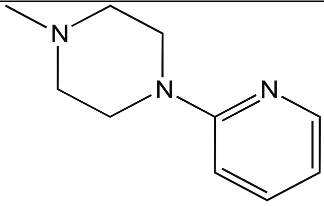	1.0	1.0	0.8
**ChX0306692**	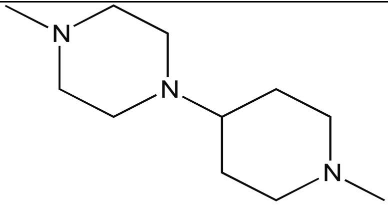	0.9	0.8	0.8

**ChX0306699**	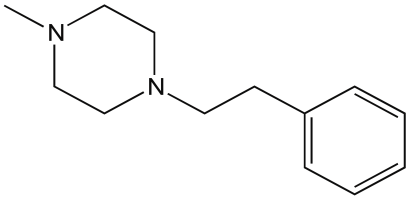	1.0	1.0	0.9
**ChX0306703**	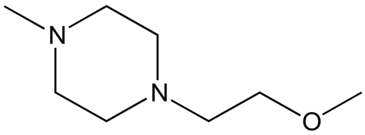	0.9	0.9	0.8
**ChX0306697**	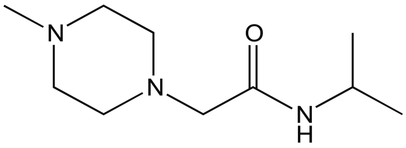	0.8	0.8	0.8
**ChX0306711**	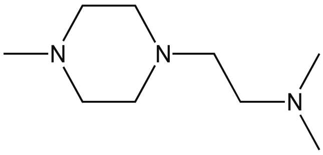	0.8	0.9	0.8
**ChX0306719**	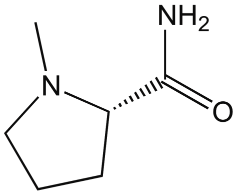	0.8	0.9	0.8
**ChX0306690**	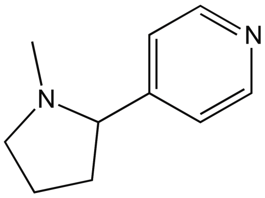	1.1	1.2	1.0
**ChX0306695**	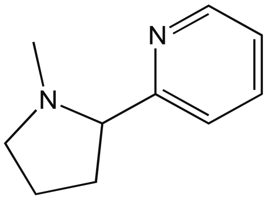	1.0	1.0	0.8
**ChX0306706**	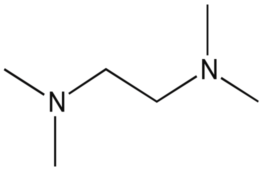	1.0	0.9	0.9

**ChX0306708**	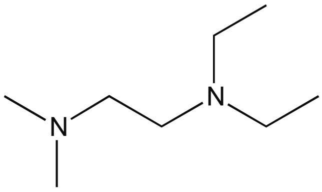	1.0	1.0	0.8
**ChX0306717**	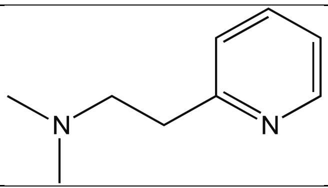	0.8	1.0	0.9
**ChX0306676**	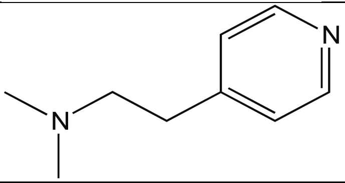	0.9	0.8	0.8

Supplementary Table S2. Chemical structures of ChX67779 analogs and corresponding ISRE-luciferase induction levels.

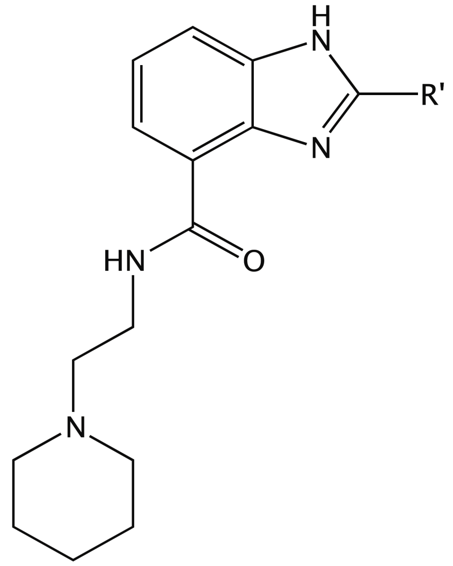

Compound ID	R’	ISRE induction fold		
		12 μM	25 μM	50 μM
**ChX0275142**	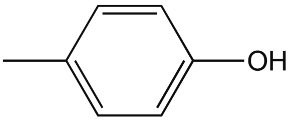	1.1	1.0	0.9
**ChX0275376**	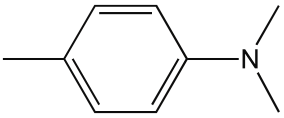	0.9	0.9	0.8

Supplementary Table S3

Primer ID	GenBank ID	Amplicon size (nt)	Sequence 5' to 3'
RPL13A-F	23521	126	CCTGGAGGAGAAGAGGAAAGAGA
RPL13A-R			TTGAGGACCTCTGTGTATTTGTCAA
IFIT1-F	3434	160	AGGACAGGAAGCTGAAGGAG
IFIT1-R			AGTGGGTGTTTCCTGCAAGG
IFIT2-F	3433	84	AATAGGACACGCTGTGGCTC
IFIT2-R			AGGCTGGCAAGAATGGAACA
ISG15-F	9636	117	CAGCGAACTCATCTTTGCCAG
ISG15-R			GACACCTGGAATTCGTTGCC
IFI6-F	2537	83	GGGTGGAGGCAGGTAAGAAA
IFI6-R			GACGGCCATGAAGGTCAGG
IFI27-F	3429	106	ATCAGCAGTGACCAGTGTGG
IFI27-R			GGCCACAACTCCTCCAATCA
IRF1-F	3659	105	TAAGGGGTGTGGCCTTTTTAGA
IRF1-R			AAAGTCAAGTTCAGGCGGGA
PKR-F	5610	158	GCGATACATGAGCCCAGAACAG
PKR-R			CTGAGATGATGCCATCCCGTAG
MxA-F	4599	74	AAGCTGATCCGCCTCCACTT
MxA-R			TGCAATGCACCCCTGTATACC
IFN-α1/13-F	3439/3447	174	CCAGTTCCAGAAGGCTCCAG
IFN-α1/13-R			TCCTCCTGCATCACACAGGC
IFN-α4-F	3441	210	CCCACAGCCTGGGTAATAGGA
IFN-α4-R			CAGCAGATGAGTCCTCTGTGC
IFN-β-F	3456	152	TGCATTACCTGAAGGCCAAGG
IFN-β-R			AGCAATTGTCCAGTCCCAGAG
IFN-γ-F	3458	177	GGCAGCCAACCTAAGCAAGAT
IFN-γ-R			CAGGGTCACCTGACACATTCA
IFN-λ1-F	282618	187	GGACGCCTTGGAAGAGTCAC
IFN-λ1-R			CTGGTCTAGGACGTCCTCCA
IFN-λ2/3-F	282616/282617	146	GGGCCTGTATCCAGCCTCAG
IFN-λ2/3-R			GAGGAGGCGGAAGAGGTTGA
